# Enantioselective Synthesis of (+)-Coerulescine by a Phase-Transfer Catalytic Allylation of Diphenylmethyl *tert*-Butyl α-(2-Nitrophenyl)Malonate

**DOI:** 10.3389/fchem.2020.577371

**Published:** 2020-11-12

**Authors:** Sangki Lee, Jewon Yang, Sehun Yang, Geumwoo Lee, Daehyun Oh, Min Woo Ha, Suckchang Hong, Hyeung-geun Park

**Affiliations:** ^1^Research Institute of Pharmaceutical Sciences, College of Pharmacy, Seoul National University, Seoul, South Korea; ^2^College of Pharmacy, Jeju National University, Jeju, South Korea

**Keywords:** coerulescine, enantioselective, synthesis, phase-transfer catalysis, alkylation

## Abstract

A 7-step enantioselective synthetic method for preparing (S)(+)-coerulescine is reported through the use of diphenylmethyl tert-butyl α-(2-nitrophenyl)malonate (16% overall yield, >99% ee). Allylation is the key step under phase-transfer catalytic conditions (86% ee). This synthetic method can be used as a practical route for the synthesis of various derivatives of (S)(+)-coerulescine for analyzing its structure–activity relationships against its biological activities.

## Introduction

**Figure 1:**
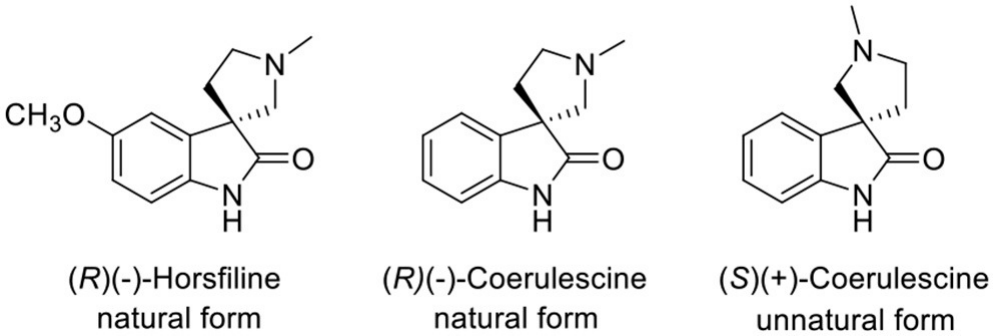
Structures of horsfiline and coerulescine.

A spirooxindole (Marti and Carreira, 2003) is a key structure of coerulescine, horsfiline, elacomine, spirotryprostatin B, and strychnofoline that has various biological activities (Galliford and Scheidt, 2007). Among the spirooxindole alkaloids, the relatively simple structure of both coerulescine and horsfiline has become attractive to many medicinal chemists as a new pharmaceutical skeleton (Figure 1). In particular, coerulescine can derivatize an aromatic moiety in oxindole via halogenation (De et al., 2015). (–)-Coerulescine was first isolated in 1998 from *Pharalis coerulescens* by Colegate et al. (Anderton et al., 1998). The construction of the quaternary stereogenic center of (–)-coerulescine has been quite challenging, and until now, only four synthetic methods have been reported for the synthesis of enantiomerically enriched coerulescine.

In 2010, Danishefsky et al. reported the synthesis of enantio-enriched (+)-coerulescine as an intermediate in the total synthesis of phalarine via an oxidative diastereoselective rearrangement of chiral tetrahydro-β-carboline precursors, which were derived from (L)-tryptophan (Li et al., 2007; Trzupek et al., 2010). In 2010, Feng et al. employed a chiral Nd(III)-*N, N*-dioxide catalyst for the α-hydroxymethylation of α-allyloxindole to introduce the quaternary chiral center (Shen et al., 2010). In 2014, Hayashi et al. confirmed the absolute configuration of coerulescine by the Michael addition of nitromethane to a 2-oxoindoline-3-ylidene acetaldehyde in the presence of a chiral diarylprolinol silyl ether (Mukaiyama et al., 2014). In 2015, Bisai et al. also constructed the quaternary stereogenic center of coerulescine via an α-hydroxymethylation of α-(aminoalkyl)oxindole using cinchona-derived thiourea bifunctional organocatalysts (De et al., 2015).

**Scheme 1:**
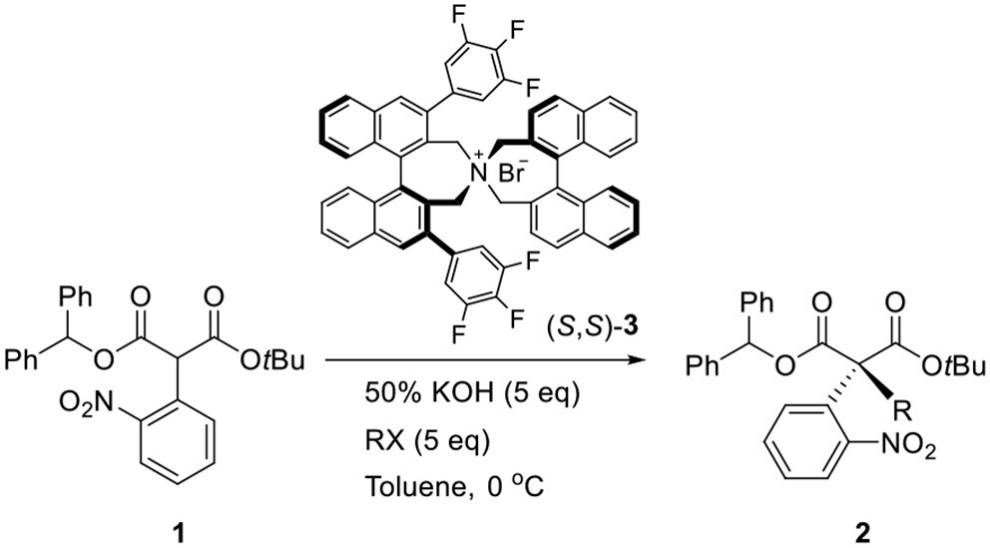
Enantioselective PTC α-alkylation of malonate.

In 2011, we reported a new synthetic method for the construction of quaternary carbon stereogenic centers from α-mono-alkylmalonates (Hong et al., 2011). Among the substrates, the asymmetric phase-transfer catalytic (PTC) α-alkylations of diphenylmethyl *tert*-butyl α-(2-nitrophenyl)malonate (**1**) in the presence of (*S*,*S*)-3,4,5-trifluorophenyl-NAS bromide (**3**) afforded the corresponding chiral quaternary α-alkylmalonate (**2**) that was successfully converted to an oxindole skeleton by the following reduction of the nitro group (Scheme 1). Given these results, we applied our methodology to synthesize (–)-horsfiline (Hong et al., 2013).

As part of our program to develop a new antitumor agent, we chose to derivatize coerulescine as a spirooxindole scaffold. The systematic investigation of the biological activity of coerulescine has not yet been performed due to the limited supply from natural isolation. In this paper, we report our efforts toward an efficient method for the synthesis of unnatural (+)-coerulescine via an enantioselective PTC (Jew and Park, 2009; Shirakawa and Maruoka, 2013) α-allylation of malonate, which is our precedent research for our studies on the structure–activity relationship and chirality–activity relationship.

## Results and Discussion

**Scheme 2:**
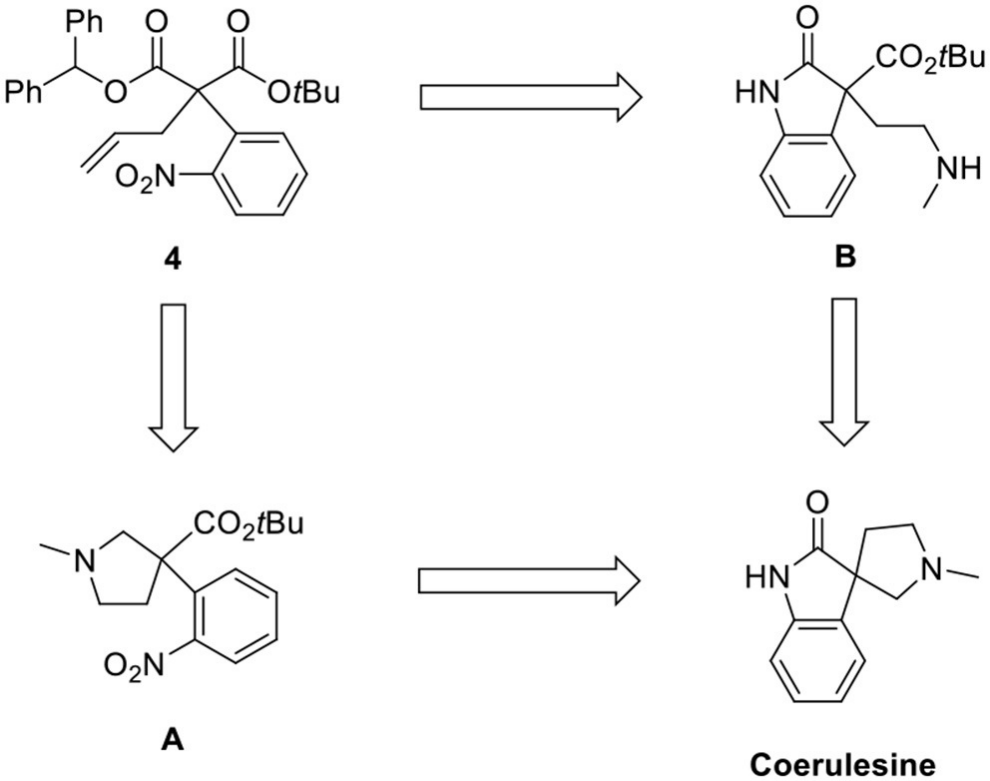
Retrosynthetic analysis.

As shown in the retrosynthetic analysis (Scheme 2), we employed two pathways [*N*-methylpyrrolidine (**A**) and oxindole (**B**)] for the construction of the spirooxindole skeleton from **4**. The corresponding methoxy-substituted *N*-methylpyrrolidine was a key intermediate for (–)-horsfiline in our previous report (Hong et al., 2013). We also attempted to convert the oxindole intermediate **B** to coerulescine by intramolecular lactamization.

First, an α-mono-aryl malonate substrate (**1**) for PTC allylation was prepared (Hong et al., 2011). A nucleophilic aromatic substitution of diphenylmethyl *tert*-butyl malonate with 2-fluoro-1-nitro-benzene under NaH base conditions in dimethylformamide (DMF) at room temperature afforded α-(2-nitrophenyl)malonate **1** (46%).

For the first step, the enantioselective allylation of **1** was performed under the reported PTC conditions (Hong et al., 2011). The PTC allylation of **1** was performed with allyl bromide (10.0 equiv.), 50% KOH (aq., 5.0 equiv.), and (*R, R*)-**3** at 0 °C in toluene to produce the allylated product (*S*)-**4** (83%, 82% ee). The optimization of the PTC conditions was achieved by varying the following parameters: solvent, base, temperature, and the amount of catalyst **3** (Table 1). Both enantioselectivity and chemical yield were dependent on the solvent and base conditions at 0 °C (entries 1–4). The highest enantioselectivity and chemical yield were observed with 50% KOH base in toluene (entry 1). In the case of temperature, lower reaction temperatures showed higher enantioselectivities (entry 1, entries 5–6). However, poor conversion was observed at −40 °C and resulted in a low chemical yield (entry 6). The optimal amount of catalyst **3** was 5 mol% (entry 5, entries 7–8). After all the tests, the following optimal allylation conditions were selected: 50% KOH aq. base and (*S, S*)-**3** (5 mol%) in toluene at −20 °C (entry 7; 87% yield, 86% ee). The enantioselectivity of the allylation of **1** in this study was slightly lower than that of α-phenyl case (Hong et al., 2011). We speculate that such a low enantioselectivity is caused by the steric effect of the *ortho*-nitro group, which may form an unfavorable binding conformation with the PTC catalyst **3**.

**Table 1:** Optimization of α-allylation under PTC conditions.

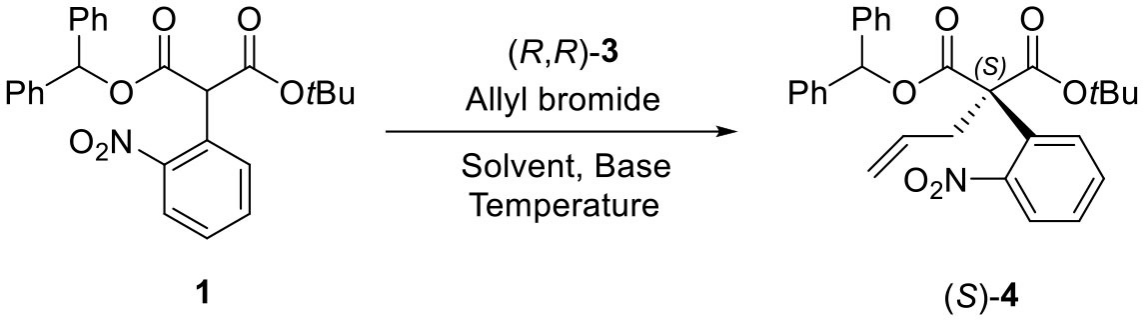							
**No**	**Solvent**	**Base**	**Cat.** **(mol%)**	**Temp. (°C)**	**Time** **(h)**	**Yield** **(%)^a^**	***Ee*** **(%)^b^**
1	Toluene	50% KOH	10	0	12	83	82
2	CH_2_Cl_2_	50% KOH	10	0	24	78	73
3	THF	50% KOH	10	0	24	75	65
4	Toluene	KOH(s)	10	0	24	67	77
5	Toluene	50% KOH	10	−20	48	87	85
6	Toluene	50% KOH	10	−40	48	52	87
7	Toluene	50% KOH	5	−20	48	87	86
8	Toluene	50% KOH	1	−20	48	75	84

a *Isolated yields*.

b *Enantiopurity was determined by an HPLC analysis of **4** using a chiral column (Chiralpak AD-H) with hexanes and 2-propanol as eluents*.

**Scheme 3:**
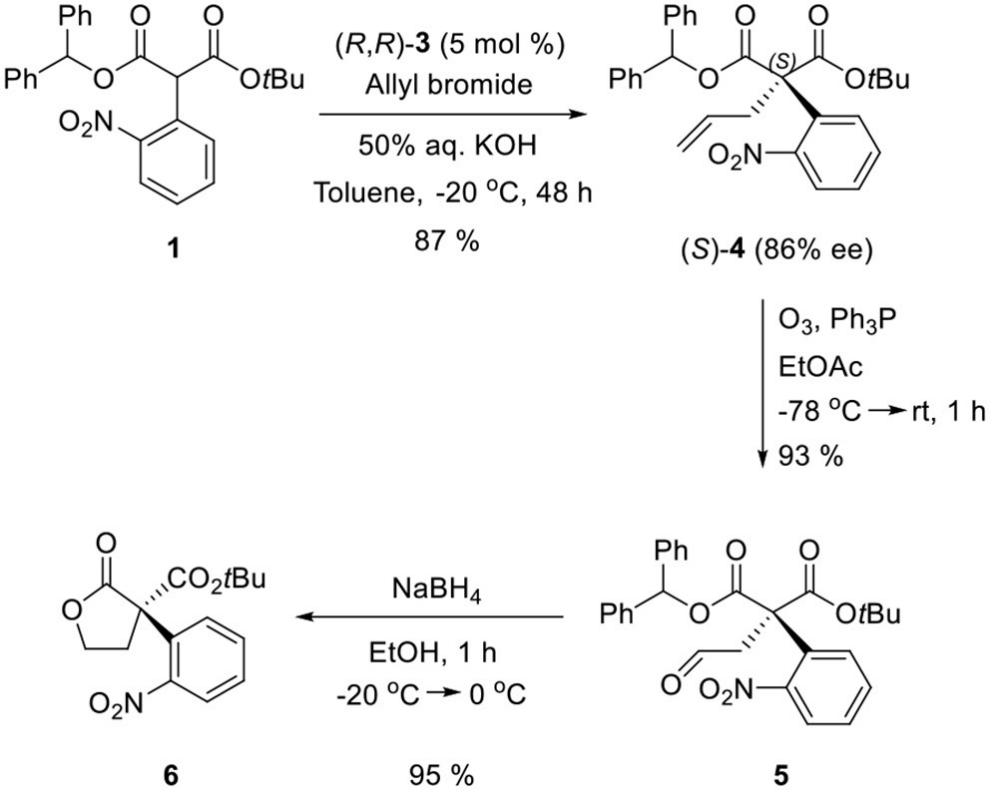
Enantioselective PTC α-allylation and conversion to lactone **6**.

**Scheme 4:**
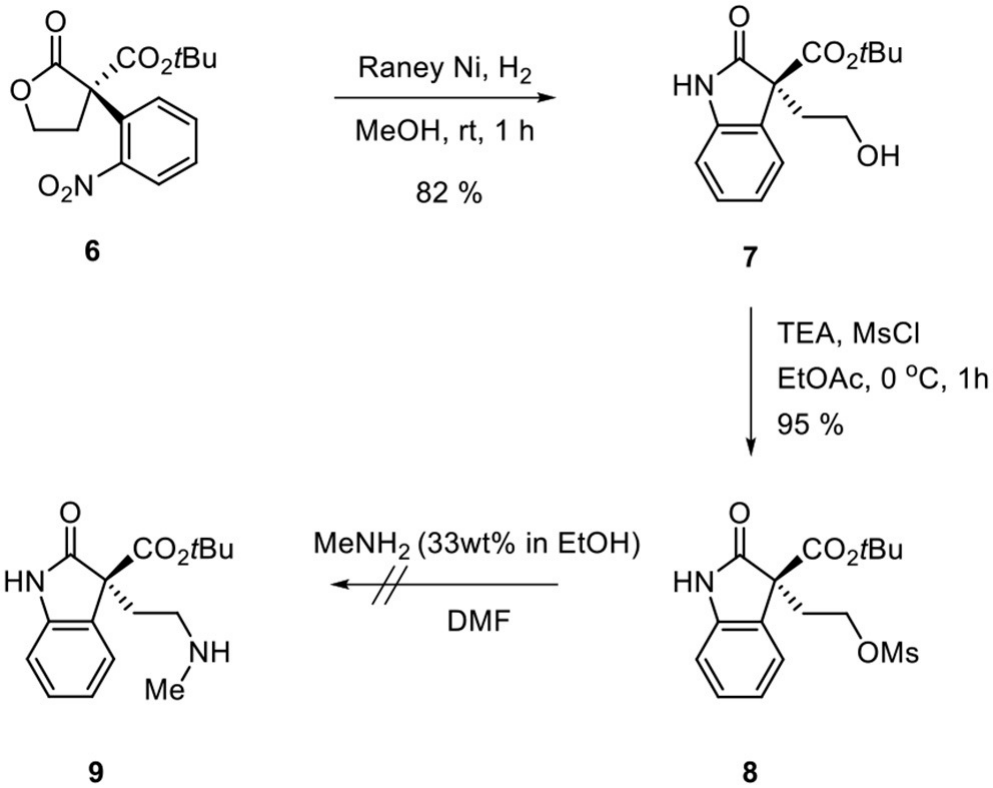
Route via oxindole **8**.

**Scheme 5:**
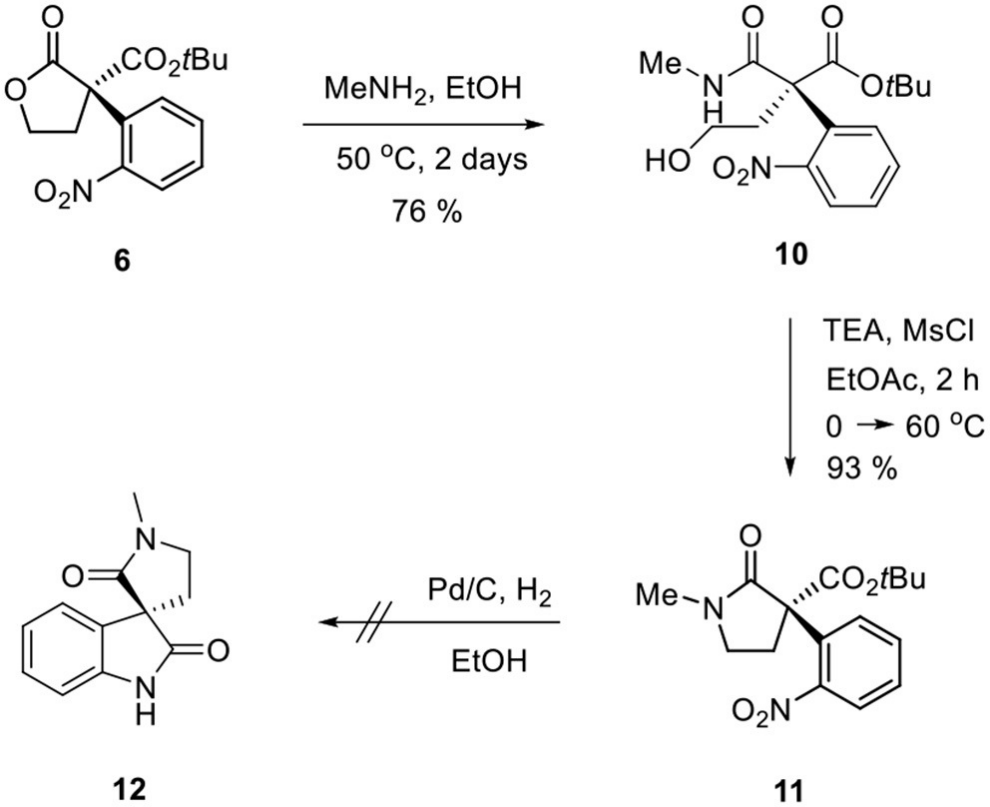
Route via *N*-methylbutyrolactam **11**.

Ozonolysis of **4**, followed by a reduction using sodium borohydride, afforded lactone **6** (95%) via aldehyde **5** (93%) (Scheme 3). Oxindole **7** was prepared by hydrogenation of **6** in the presence of Raney Ni/H_2_ and the following mesylation (**8**) under trimethylamine basic conditions (Scheme 4). However, the treatment of methylamine (33 wt % in absolute EtOH) in various solvents such as DMF, CH_3_CN, CH_2_Cl_2_, and DMSO at room temperature did not give the expected amine product **9** or the further cyclized *N*-methylpyrrolidine analog **12**. Only the starting material **8** was recovered. The increase of temperature led to so many side products. By another route, we tried to prepare *N*-methylbutyrolactam **12**, which could be selectively reduced to produce coerulescine (Scheme 5) (Trost and Brennan, 2006). The ring opening of lactone **6** with *N*-methylamine afforded *N*-methylamide **10**. The mesylation of **10** followed by an intramolecular lactamization successfully provided **11**. However, the catalytic hydrogenation under Pd/C, Pd(OH)_2_, PtO_2_, and Raney Ni under H_2_ (1 atm) gave no oxindole **12**. We speculate that the unsuccessful results were due to the carbonyl group in lactam moiety.

**Scheme 6:**
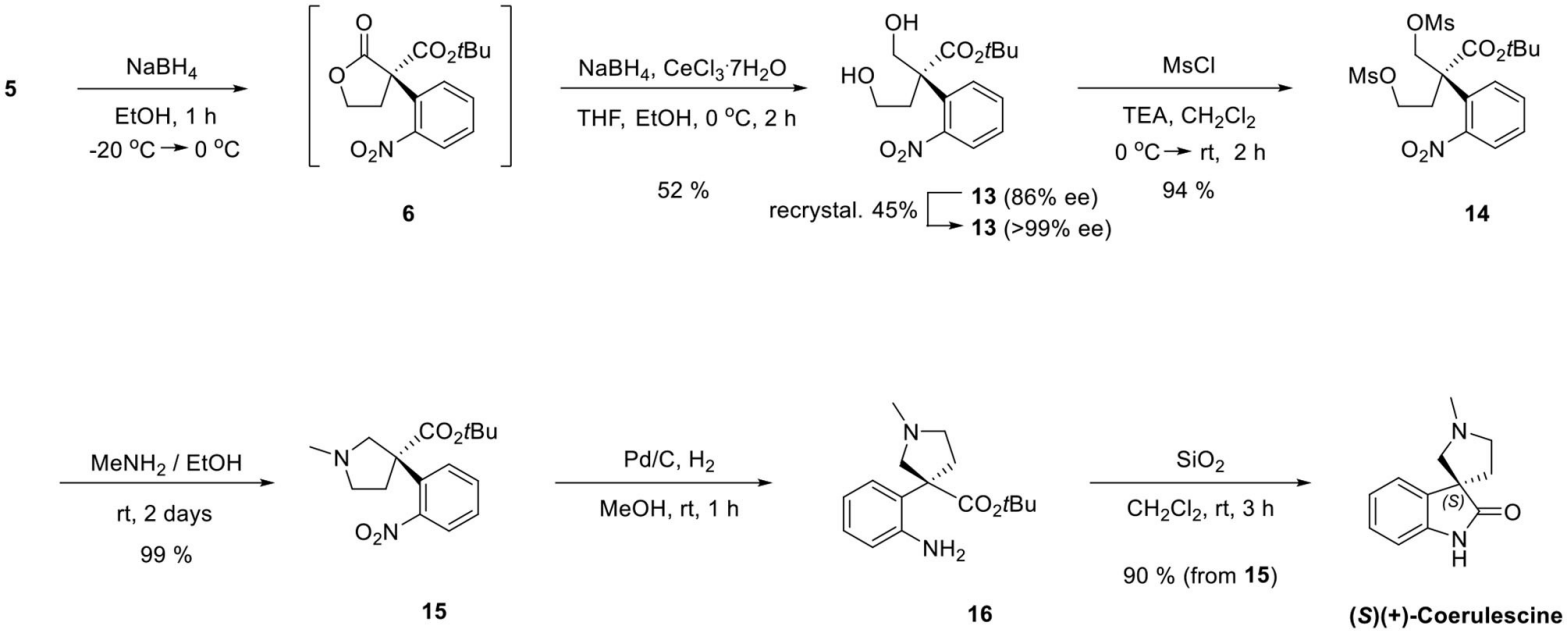
Completion of total synthesis of (+)-coerulescine.

Finally, we adapted our previous synthetic route of horsfiline, as shown in Scheme 6. Without purifying **6**, which could be prepared from **5**, the treatment of additional sodium borohydride with cerium (III) trichloride heptahydrate and tetrahydrofuran as a cosolvent at 0 °C selectively reduced **6** to the corresponding diols **13** (52% from **5**) *in situ* (Martin et al., 2010). Diol **13** was purified as a single stereoisomer (>99% ee) with a 45% yield by recrystallizing (86% ee) using ethylacetate and hexane (1:5). Dimesylation of **13** (94%) followed by *N*-alkylation followed by intramolecular *N*-alkylation in the presence of excess methylamine successfully produced *N*-methylpyrrolidines **15** (99%). The reduction of the nitro group on **15** by a catalytic hydrogenation under Pd/C and atmospheric H_2_ afforded amine **16**. Finally, the prepared amine **16** was directly cyclized to (+)-coerulescine {observed ${\text{[α]}}_{\text{D}}^{20}$ = 3.08 (*c* 1, MeOH); literature ${\text{[α]}}_{\text{D}}^{20}$ = 1.0 (*c* 2.4, MeOH) (Mukaiyama et al., 2014)} by stirring with silica gel (SiO_2_) in CH_2_Cl_2_ with no racemization (90% from **15**, >99% ee).

## Conclusion

In summary, as a precedent study for systematic research of biological activities, enantioselective synthetic routes of (+)-coerulescine were investigated. (+)-Coerulescine was prepared through seven steps from diphenylmethyl *tert*-butyl α-(2-nitrophenyl)malonate via an enantioselective PTC allylation as the key step (16% overall yield, >99% ee). The large scalable synthetic method of coerulescine enables a systematic investigation of its antitumor activity. Further preparation of derivatives of coerulescine is now under investigation for structure–activity relationship studies.

## Materials and Methods

### Experimental Section

#### General Information

All reagents purchased from commercial sources were used without further purification. The phase-transfer catalyst, (*R*,*R*)-3,4,5-trifluorophenyl-NAS bromide (**3**), was purchased from commercial sources. TLC analyses were performed using pre-coated TLC plate (silica gel 60 GF254, 0.25 mm). Flash column chromatography was performed on flash silica gel 230–400 mesh size. The values of enantiomeric excess (*ee*) of chiral products were determined by HPLC using 4.6 × 250 mm DAICEL Chiralpak AD-H and Chiralpak AS-H. Infrared analyses (neat) were performed by FT-IR. ^1^H-NMR spectra were recorded at 400 MHz with reference to CHCl_3_ (δ 7.24). ^13^C-NMR spectra were obtained by 100 MHz spectrometer relative to the central CDCl_3_ (δ 77.0) resonance. Coupling constants (*J*) in ^1^H-NMR are in hertz. Low-resolution mass spectra and high-resolution mass spectra (HRMS) were measured on positive-ion FAB spectrometer. Melting points were measured on melting point apparatus and were uncorrected. Optical rotations were measured on a polarimeter and calibrated with pure solvent as blank.

#### Synthetic Methods of Materials

##### 1-Benzhydryl 3-(tert-butyl) 2-(2-nitrophenyl)malonate (1)

Sodium hydride (19.4 mg, 0.81 mmol) was added to a stirred solution of diphenylmethyl *tert*-butyl malonate (202 mg, 0.62 mmol) in DMF (3 ml) at 0 °C. After stirring for 30 min, 2-fluoronitrobenzene (0.065 ml, 0.62 mmol) (Hong et al., 2011) was slowly added to the reaction mixture and stirred for 48 h. The reaction mixture was evaporated and diluted with EtOAc (60 ml), quenched with ammonium chloride (20 ml), washed with brine (20 ml), dried over anhydrous MgSO_4_, filtered, and concentrated *in vacuo*. The residue was purified by column chromatography (silica gel, hexanes:EtOAc = 10:1) to afford **1** (127.5 mg, 46% yield) as a pale yellow solid. m.p = 111.6 °C; ^1^H-NMR (300 MHz, CDCl_3_) δ 8.03 (dd, *J*_1_ = 7.86 Hz, *J*_2_ = 1.47 Hz, 1H), 7.54–7.43 (m, 3H), 7.37–7.28 (m, 12H), 6.96 (s, 1H), 5.33 (s, 1H), 1.39 (s, 9H) ppm; ^13^C-NMR (100 MHz, CDCl_3_) δ 166.39, 165.59, 148.57, 139.05, 133.05, 130.83, 128.80, 128.32, 128.23, 128.21, 128.10, 127.87, 126.98, 124.76, 82.97, 78.35, 55.28, 27.43 ppm; IR (KBr) 2,980, 1,732, 1,579, 1,529, 1,496, 1,455, 1,394, 1,348, 1,306, 1,228, 1,142, 1,001, 854, 786, 744, 700 cm^−1^; HRMS (FAB): calcd for [C_26_H_26_O_6_N]^+^: 448.1760, found: 448.1753. The spectral data were identical with the reported data (Hong et al., 2011).

##### (S)-1-Benzhydryl 3-tert-butyl 2-allyl-2-(2-nitrophenyl) malonate (4)

Allylic bromide (0.096 ml, 1.11 mmol) was added to a solution of **1** (100 mg, 0.22 mmol) and (*R*,*R*)-3,4,5-trifluorophenyl-NAS bromide (**3**, 10.2 mg, 0.011 mmol) in toluene (0.75 ml). At −20 °C, aq. 50% KOH (0.125 ml, 1.11 mmol) was added to the reaction mixtures. EYELA PSL-1400 was used for low-temperature stirring and the stirring rate was 7, and commercially available KOH pellet (99%) was grinded to prepare solid KOH as powder form. 50% *w*/*v* aqueous KOH was used as stock solution. After stirring for 48 h, the reaction mixture was diluted with EtOAc (30 ml), washed with brine (2 × 15 ml), dried over anhydrous MgSO_4_, filtered, and concentrated *in vacuo*. The residue was purified by column chromatography (silica gel, hexane:EtOAc = 20:1) to afford **4** as a white solid (95 mg, 87% yield). The enantioselectivity was determined by chiral HPLC analysis (DAICEL Chiralpak AD-H, hexane:2-propanol = 95:5), flow rate = 1.0 ml/min, 23 °C, λ = 254 nm, retention time, *R* (minor) 11.5 min, *S* (major) 15.8 min, 86% *ee*. ${\text{[α]}}_{\text{D}}^{20}$ = −30.3 (86% *ee, c* = 1, CHCl_3_), m.p = 88.9 °C; ^1^H-NMR (400 MHz, CDCl_3_) δ 7.96–7.99 (m, 1H), 7.39–7.43 (m, 2H), 7.22–7.30 (m, 10H), 7.08–7.10 (m, 1H), 6.94 (s, 1H), 5.61–5.71 (m, 1H), 4.85–4.96 (m, 2H), 3.27–3.29 (m, 2H), 1.27 (s, 9H) ppm; ^13^C-NMR (100 MHz, CDCl_3_) δ 168.39, 167.00, 149.37, 139.33, 139.27, 133.23, 132.35, 132.15, 131.25, 128.33, 128.31, 128.23, 127.98, 127.94, 127.41, 127.20, 125.55, 118.86, 83.53, 78.46, 63.85, 39.57, 27.46 ppm; IR (KBr) 2,980, 1,733, 1,532, 1,454, 1,358, 1,252, 1,205, 1,184, 1,147, 986, 855, 744, 700 cm^−1^; HRMS (FAB): calcd for [C_29_H_30_O_6_N]^+^: 488.2073, found: 488.2091.

##### (S)-1-Benzhydryl 3-tert-butyl 2-(5-methoxy-2-nitrophenyl) -2-(2-oxoethyl)malonate (5)

A solution of **4** (538 mg, 1.1 mmol) in EtOAc (17 ml) was purged with O_3_ at −78 °C until no more starting material was observed by TLC analysis (2 min). The excess O_3_ in the solution was removed by purging of argon at −78 °C for 1 h 30 min. To the mixture, four equivalents of triphenyl phosphine (1.2 g, 4.4 mmol) were added at −78 °C, and then stirred at −78 °C to room temperature for 20 min. After stirring for 2 h, all solvent was removed on a rotary evaporator. Also, the residue was purified by column chromatography (silica gel, hexane:EtOAc = 5:1) to afford **5** (501 mg, 93% yield) as a pale yellow viscous oil. ${\text{[α]}}_{\text{D}}^{20}$ = −105.2 (86% *ee, c* =1, CHCl_3_); ^1^H-NMR (400 MHz, CDCl_3_) δ 9.67–9.68 (m, 1H), 8.01 (dd, *J*_1_ = 8.0, *J*_2_ = 1.1 Hz, 1H), 7.46 (td, *J*_1_ = 7.8 Hz, *J*_2_ = 1.4 Hz, 1H), 7.37 (td, *J*_1_ = 7.5 Hz, *J*_2_ = 1.4 Hz, 1H), 7.24–7.30 (m, 10H), 7.00–7.02 (m, 2H), 3.35–3.58 (m, 2H), 1.26 (s, 9H) ppm; ^13^C-NMR (100 MHz, CDCl_3_) δ 198.50, 167.86, 166.13, 148.84, 138.56, 138.42, 132.94, 131.49, 130.44, 129.04, 128.38, 128.30, 128.15, 127.53, 127.08, 126.12, 84.89, 79.46, 62.08, 48.44, 27.23 ppm; IR (KBr) 3,033, 2,980, 2,933, 2,755, 1,727, 1,533, 1,455, 1,357, 1,268, 1,212, 1,147, 1,079, 956, 856, 744, 701 cm^−1^; HRMS (ESI): calcd for [C_28_H_27_NO_7_Na]^+^: 512.1680, found: 512.1702.

##### (S)-tert-Butyl 3-(2-nitrophenyl)-2-oxotetrahydrofuran -3-carboxylate (6)

Sodium borohydride (45.4 mg, 1.2 mmol) was added to a solution of **5** (519.5 mg, 1 mmol) in ethanol (6 ml) at −20 °C. The reaction mixture was stirred for 1 h until entire substrate was converted into 6 by TLC analysis. 1N-NaOH (3–4 drops) was added dropwise to the reaction mixture for quenching extra sodium borohydride. After all solvent was removed on a rotary evaporator, the mixture was diluted with EtOAc (20 ml) and brine (20 ml). The layers were separated and the aqueous layer was extracted with EtOAc (2 × 20 ml). The combined organic layers were dried with MgSO_4_, and concentrated *in vacuo*. The residue was purified by column chromatography (silica gel, hexane:EtOAc = 5:1–3:1) to afford **6** as white solid (313.5 mg, 93% yield). m.p = 109.8 °C; ${\text{[α]}}_{\text{D}}^{20}$ = −55.77 (86% ee, *c* = 1, CHCl_3_); ^1^H NMR (400 MHz, CDCl_3_)δ 8.11 (dd, *J*_2_ = 1.40 Hz, 1H), 7.63 (td, *J*_1_ = 7.50 Hz, *J*_2_ = 1.40 Hz, 1H), 7.51 (td, *J*_1_ = 7.80 Hz, *J*_2_ = 1.30 Hz, 1H), 7.37 (dd, *J*_1_ = 7.80Hz, *J*_2_ = 1.40Hz, 1H), 4.58 (td, *J*_1_ = 8.60 Hz, *J*_2_ = 4.90 Hz, 1H), 4.22–4.28 (m, 1H), 3.59–3.66 (m, 1H), 2.56 (qd, *J*_1_ = 7.00 Hz, *J*_2_ = 4.60 Hz, 1H), 1.39 (s, 9H) ppm; ^13^C-NMR (100 MHz, CDCl_3_) δ 173.76, 165.93, 148.23, 133.84, 132.75, 129.72, 129.06, 126.14, 84.39, 66.48, 61.38, 36.25, 27.42 ppm; IR (KBr) 2,981, 1,776, 1,731, 1,609, 1,577, 1,531, 1,482, 1,456, 1,371, 1,354, 1,255, 1,220, 1,158, 1,108, 1,030, 963, 857, 840, 772, 707, 684 cm^−1^; HRMS (FAB): calcd for [C_15_H_17_NO_6_]^+^: 307.1056, found: 308.1126.

##### (S)-tert-Butyl 3-(2-hydroxyethyl)-2-oxoindoline-3- carboxylate (7)

Raney Nickel (202 mg) was added to a stirred solution of **6** (67.5 mg, 0.2 mmol) in MeOH (3 ml) under H_2_ gas (1 atm) and stirred for 1 h. The reaction mixtures were filtered through the Celite 545 and concentrated *in vacuo*. The residue was purified by column chromatography (silica gel, hexane:EtOAc = 1:1–1:2) to afford **7** as white solid (50.4 mg, 82% yield). m.p = 118.2 °C; ${\text{[α]}}_{\text{D}}^{20}$ = +111.42 (86% ee, *c* = 1, CHCl_3_); ^1^H-NMR (400 MHz, CDCl_3_) δ 9.01 (s, 1H), 7.20 −7.25 (m, 2H), 7.02 (t, *J* = 7.50 Hz, 1H), 6.88–6.91 (m, 1H), 3.71 (q, *J* = 5.50 Hz, 1H), 3.55 (t, *J* = 5.50 Hz, 1H), 2.92 (s, 1H), 2.57 (qd, *J*_1_ = 7.40 Hz, *J*_2_ = 5.30 Hz, 1H), 2.28 (dt, *J*_1_ = 14.3 Hz, *J*_2_ = 5.50 Hz, 1H) 1.35 (s, 9H) ppm; ^13^C-NMR (100 MHz, CDCl_3_) δ 177.92, 168.10, 141.25, 128.96, 128.80, 123.42, 122.71 110.33, 82.80, 59.61, 58.92, 36.51, 27.65 ppm; IR (KBr) 3,249, 1,733, 1,620, 1,472, 1,369, 1,220, 1,155, 772, 674 cm^−1^; HRMS (FAB): calcd for [C_15_H_19_NO_4_]^+^: 277.1314, found: 278.1397.

##### (S)-tert-Butyl 3-(2-((methylsulfonyl)oxy)ethyl) -2-oxoindoline -3-carboxylate(8)

Methanesulfonyl chloride (0.058 ml, 0.75 mmol) was added to a solution of **7** (153.7 mg, 0.5 mmol) and triethylamine (0.209 ml, 1.5 mmol) in EtOAc (2 ml) at 0 °C. The reaction mixture was allowed to warm to rt and stirred for 1 h. Saturated aqueous solution of NaHCO_3_ (10 ml) was added to the reaction mixture for quenching and the mixture was diluted with EtOAc (50 ml). The organic layers were separated and washed with brine (20 ml), dried with MgSO_4_, and concentrated *in vacuo*. The residue was purified by column chromatography (silica gel, hexane:EtOAc = 3:1–1:1) to afford **8** as yellow oil (183 mg, 95% yield). ${\text{[α]}}_{\text{D}}^{20}$ = −41.36 (86% ee, *c* = 0.5, CHCl_3_); ^1^H-NMR (400 MHz, CDCl_3_) δ 8.48 (s, 1H), 7.27−7.29 (m, 1H), 7.20–7.22 (d, *J* = 7.3 Hz, 1H), 7.03–7.07 (td, *J*_1_ = 7.5 Hz, *J*_2_ = 0.9 Hz, 1H), 6.92–6.94 (d, *J* = 3.9 Hz, 1H), 4.20–4.25 (m, 1H), 4.09–4.15 (m, 1H), 2.81 (s, 3H), 2.72–2.78 (q, *J* = 7.2 Hz, 1H), 2.52–2.59 (qd, *J*_1_ = 7.0 Hz, *J*_2_ = 5.3 Hz, 1H), 1.35 (s, 9H) ppm; ^13^C-NMR (100 MHz, CDCl_3_) δ 175.60, 167.22, 141.25, 129.40, 127.71, 123.47, 122.93, 110.29, 83.24, 65.60, 58.38, 37.07, 32.37, 27.62 ppm; IR (KBr) 1,736, 1,619, 1,472, 1,356, 1,220, 1,174, 934, 772, 674 cm^−1^; HRMS (FAB): calcd for [C_16_H_21_NO_6_S]^+^: 355.1090, found: 355.1089.

##### (S)-tert-Butyl 4-hydroxy-2-(methylcarbamoyl)-2-(2- nitrophenyl)butanoate (10)

**6** (202.4 mg, 0.6 mmol) was dissolved in methylamine solution 33 wt % in absolute ethanol (5 ml) under argon gas. At 50 °C, the reaction mixture was stirred for 48 h. After all solvent was removed on a rotary evaporator, the residue was purified by column chromatography (silica gel, hexane:EtOAc = 1:2–only EtOAc) to afford **10** as a yellow solid (168 mg, 76% yield). m.p = 113.4 °C; ${\text{[α]}}_{\text{D}}^{20}$ = −90.75 (86% ee, *c* = 1, CHCl_3_); ^1^H-NMR (400 MHz, CDCl_3_) δ 8.02 (dd,*J*_1_ = 8.00 Hz, *J*_2_ = 1.10 Hz, 1H), 7.54–7.63 (m, 2H), 7.42–7.46 (m, 1H), 3.58–3.73 (m, 2H), 2.82 (d, *J* = 5.00 Hz, 3H), 2.72 (dt, *J*_1_ = 14.3 Hz, *J*_2_ = 6.3 Hz, 1H), 2.55–2.62 (m, 2H), 1.30 (s, 9H) ppm; ^13^C-NMR (100 MHz, CDCl_3_) δ 171.85, 170.51, 148.48, 133.99, 132.94, 130.19, 128.16, 125.37, 83.97, 60.68, 59.06, 38.94, 27.43, 26.55 ppm; IR (KBr) 3,368, 2,977, 1,714, 1,658, 1,526, 1,479, 1,457, 1,413, 1,395, 1,370, 1,352, 1,254, 1,220, 1,154, 1,048, 856, 837, 772, 713, 673 cm^−1^; HRMS (FAB): calcd for [C_16_H_22_N_2_O_6_]^+^: 338.1478, found: 339.1558.

##### (S)-tert-Butyl 1-methyl-3-(2-nitrophenyl)-2-oxopyrrolidine -3-carboxylate (11)

Methanesulfonyl chloride (0.093 ml, 1.2 mmol) was added to a solution of **10** (368.4 mg, 1 mmol) and triethylamine (0.347 ml, 2.5 mmol) in EtOAc (4 ml) at 0 °C. The reaction mixture was allowed to warm to 60 °C and stirred for 2 h. Saturated aqueous solution of NaHCO_3_ (15 ml) was added dropwise to the reaction mixture for quenching and the mixture was diluted with EtOAc (20 ml). The organic layers were separated and the aqueous layer was extracted with dichloromethane (20 ml). The combined organic layers were dried with MgSO_4_ and concentrated *in vacuo*. The residue was purified by column chromatography (silica gel, hexane:EtOAc = 5:1–3:1) to afford **11** as a pale yellow oil (325.8 mg, 93% yield). ${\text{[α]}}_{\text{D}}^{20}$ = −23.42 (86% ee, *c* = 1, CHCl_3_); ^1^H-NMR (400 MHz, CDCl_3_) δ 8.06 (d, *J* = 7.80 Hz, 2H), 7.63–7.67 (m, 1H), 7.47 (t, *J* = 8.00 Hz, 2H), 3.43–3.55 (m, 2H), 2.82 (d, *J* = 4.60 Hz, 3H), 2.75–2.93 (m, 2H), 1.30 (s, 9H) ppm; ^13^C-NMR (100 MHz, CDCl_3_) δ 171.09, 168.89, 148.37, 133.53, 133.26, 129.26, 128.35, 125.64, 84.29, 60.96, 39.91, 39.54, 27.43, 26.50 ppm; IR (KBr) 3,370, 2,978, 2,929, 1,714, 1,669, 1,529, 1,458, 1,412, 1,395, 1,369, 1,354, 1,279, 1,254, 1,219, 1,153, 1,068, 855, 839, 772, 742, 711 cm^−1^; HRMS (FAB): calcd for [C_16_H_20_N_2_O_5_]^+^: 320.1372, found: 321.1451.

##### (S)-tert-Butyl 4-hydroxy-2-(hydroxymethyl)-2-(2 -nitrophenyl)butanoate (13)

Sodium borohydride (47 mg, 1.2 mmol) was added to a solution of **5** (488 mg, 0.99 mmol) in ethanol (6.6 ml) at −20 °C. After stirring for 1 h, the reaction mixture allowed to warm to 0 °C until the complete conversion of substrate into intermediate **6** by TLC analysis. Tetrahydrofuran (1.6 ml) and cerium(III) trichloride heptahydrate (742 mg, 1.99 mmol) were added at 0 °C. After stirring the reaction mixture for 10 min at 0 °C, sodium borohydride (188 mg, 4.98 mmol) was added and stirred for 10 min then a second charge of sodium borohydride (188 mg, 4.98 mmol) was added. The reaction mixture was stirred for 2 h until no more intermediate 7 was observed by TLC analysis, then AcOH (0.5 ml) was added dropwise for quenching the extra sodium borohydride. After all of solvent was removed *in vacuo*, the reaction mixture was diluted with ethyl acetate (20 ml) and of water (20 ml). The organic layers were separated and the aqueous layer was extracted with ethyl acetate (2 × 20 ml). The combined organic layers were washed with a saturated aqueous solution of NaHCO_3_ (20 ml) and brine (20 ml), dried with MgSO_4_, and concentrated *in vacuo*. The residue was purified by column chromatography (silica gel, hexane:EtOAc = 1:1 to only EtOAc) to afford **13** (161 mg, 52% yield) as a white solid. The obtained **13** was recrystallized three times sequentially with hexane–ethyl acetate (5:1) to afford the S-enantiomer **13** (>99% *ee*, 74 mg, 45%) as a single stereoisomer. The enantioselectivity was determined by chiral HPLC analysis (DAICEL Chiralpak AD-H, hexane:2-propanol = 90:10), flow rate = 1.0 ml/min, 23 °C, λ = 254 nm, retention time, *S* (major) 16.2 min, *R* (minor) 18.7 min, >99% *ee*. ${\text{[α]}}_{\text{D}}^{20}$ = +110.8 (>99% *ee, c* = 1, CHCl_3_), m.p = 140.8 °C; ^1^H-NMR (400 MHz, CDCl_3_) δ 7.88 (d, *J* = 8.2 Hz, 1H), 7.58–7.69 (m, 2H), 7.42 (t, *J* = 7.5 Hz, 1H), 4.36 (d, *J* = 11.9 Hz, 1H), 3.94 (d, *J* = 11.4 Hz, 1H), 3.77–3.83 (m, 1H), 3.62 (q, *J* = 5.6 Hz, 1H), 3.28 (s, 1H), 2.39–2.57 (m, 2H), 2.31 (s, 1H), 1.41 (s, 9H) ppm; ^13^C-NMR (100 MHz, CDCl_3_) δ 172.21, 149.90, 134.33, 132.74, 129.99, 128.07, 125.63, 83.53, 67.79, 59.04, 55.05, 37.81, 27.71 ppm; IR (KBr) 3,373, 2,979, 1,718, 1,529, 1,357, 1,250, 1,157, 1,038, 853, 841, 787, 748, 712 cm^−1^; HRMS (FAB): calcd for [C_15_H_22_NO_6_]^+^: 312.1447, found: 312.1434.

##### (S)-tert-Butyl 2-(2-nitrophenyl)-2- (methanesulfonyloxymethyl)-4-methanesulfonyl -oxybutanoate (14)

Methanesulfonyl chloride (46 μl, 0.6 mmol) was added dropwise to a solution of **13** (62 mg, 0.2 mmol) and triethylamine (209 ml, 1.5 mmol) in dichloromethane (3 ml) at 0 °C. The reaction mixture was allowed to warm to room temperature and stirred for 10 min. Saturated aqueous solution of NaHCO_3_ (10 ml) was added dropwise to the reaction mixture for quenching and the mixture was diluted with dichloromethane (20 ml). The layers were separated and the aqueous layer was extracted with dichloromethane (2 × 20 ml). The combined organic layers were washed with water (20 ml) and brine (20 ml), dried with MgSO_4_, and concentrated *in vacuo*. The residue was purified by column chromatography (silica gel, hexane:EtOAc = 1:1) to afford **14** (88 mg, 94% yield) as a colorless oil. ${\text{[α]}}_{\text{D}}^{20}$ = +118.4 (>99% *ee, c* = 1, CHCl_3_); ^1^H-NMR (400 MHz, CDCl_3_) δ 7.95–7.98 (m, 1H), 7.65–7.69 (m, 1H), 7.50–7.54 (m, 2H), 4.96 (d, *J* = 10.1 Hz, 1H), 4.76 (d, *J* = 10.5 Hz, 1H), 4.10–4.23 (m, 2H), 2.95 (s, 3H), 2.85 (s, 3H), 2.62–2.84 (m, 2H), 1.45 (s, 9H) ppm; ^13^C-NMR (100 MHz, CDCl_3_) δ 168.45, 149.77, 132.92, 130.84, 130.25, 129.13, 126.02, 84.85, 71.98, 65.47, 52.83, 37.40, 37.19, 33.67, 31.49, 27.64 ppm; IR (KBr) 2,981, 2,940, 1,725, 1,533, 1,461, 1,359, 1,256, 1,176, 961, 836, 789, 748 cm^−1^; HRMS (FAB): calcd for [C_17_H_26_NO_10_S_2_]^+^: 468.0998, found: 468.0986.

##### (S)-tert-Butyl 3-(2-nitrophenyl)-1-methylpyrrolidine-3- carboxylate (15)

Compound **14** (82 mg, 0.17 mmol) was dissolved in methylamine solution [33 wt % in absolute ethanol (4.4 ml)]. At room temperature, the reaction mixture was stirred for 48 h. After all of the solvent was removed *in vacuo*, the residue was purified by column chromatography (silica gel, hexane:EtOAc:acetone:methanol = 20:16:4:1) to afford **15** (54 mg, 99% yield) as a yellow oil. ${\text{[α]}}_{\text{D}}^{20}$ = −114.1 (>99% *ee, c* = 1, CHCl_3_); ^1^H-NMR (400 MHz, CDCl_3_) δ 7.88 (dd, *J*_1_ = 8.3 Hz, *J*_2_ = 1.4 Hz, 1H), 7.77 (dd, *J*_1_ = 8.3 Hz, *J*_2_ = 0.9 Hz, 1H), 7.55–7.59 (m, 1H), 7.33–7.38 (m, 1H), 3.33 (d, *J* = 10.1 Hz, 1H), 3.02 (d, *J* = 5.5 Hz, 1H), 2.90–2.96 (m, 1H), 2.82 (d, *J* = 10.1 Hz, 1H), 2.55 (q, *J* = 8.6 Hz, 1H), 2.42 (s, 3H), 2.18 (s, 1H), 1.37 (s, 9H) ppm; ^13^C-NMR (100 MHz, CDCl_3_) δ 172.65, 148.21, 139.93, 133.05, 129.01, 127.17, 124.70, 81.75, 67.17, 57.22, 56.44, 41.84, 38.56, 27.55 ppm; IR (KBr) 2,976, 2,939, 2,841, 2,788, 1,731, 1,607, 1,575, 1,528, 1,479, 1,455, 1,357, 1,251, 1,154, 1,085, 1,045, 897, 853, 787, 749, 712 cm^−1^; HRMS (FAB): calcd for [C_16_H_23_N_2_O_4_]^+^: 307.1658, found: 307.1656.

##### (S)(+)-Coerulescine

Pd/C (11 mg) was added to a stirred solution of **15** (45 mg, 0.15 mmol) in methanol (3 ml) under H_2_ gas and stirred for 1 h. The reaction mixture was filtered through the Celite 545 and concentrated *in vacuo* to afford quantitative **16** as a yellow oil. Silica gel (545 mg) was added to a stirred solution of **16** in dichloromethane (1.4 ml) and stirred for 3 h. The absorbed residue was purified by column chromatography (silica gel, dichloromethane/methanol = 20:1–8:1) to afford (*S*)-(+)-coerulescine (27 mg, 90% yield) as a white solid. Absolute configuration was determined by comparing the HPLC data with the literature of Hayashi group (Mukaiyama et al., 2014). The enantioselectivity was determined by chiral HPLC analysis (DAICEL Chiralpak AS-H, hexane:2-propanol = 90:10), flow rate = 1.0 ml/min, 23 °C, λ = 254 nm, retention time, *R* (minor) 30.1 min, *S* (major) 48.9 min, >99% *ee*. ${\text{[α]}}_{\text{D}}^{20}$ = +3.08 (>99% *ee*, c = 1, MeOH), Lit. (Mukaiyama et al., 2014) ${\text{[α]}}_{\text{D}}^{20}$ = +1.0 (99% *ee, c* = 2.4, MeOH), m.p = 122.6 °C; ^1^H-NMR (400 MHz, CDCl_3_) δ 8.69 (s, 1H), 7.38 (d, *J* = 6.9 Hz, 1H), 7.18 (td, *J*_1_ = 7.7 Hz, *J*_2_ = 1.1 Hz, 1H), 7.01–7.05 (m, 1H), 6.88 (d, *J* = 7.8 Hz, 1H), 3.00 (td, *J*_1_ = 8.0 Hz, *J*_2_ = 5.1 Hz, 1H), 2.76–2.90 (m, 3H), 2.46 (s, 3H), 2.37–2.44 (m, 1H), 2.06–2.13 (m, 1H) ppm; ^13^C-NMR (100 MHz, CDCl_3_) δ 183.47, 140.36, 136.11, 127.67, 123.11, 122.67, 109.70, 66.26, 56.73, 53.70, 41.80, 37.85 ppm; IR (KBr) 3,208, 2,925, 2,850, 2,790, 1,712, 1,620, 1,471, 1,337, 1,244, 1,198, 1,153, 1,104, 1,015, 754, 677 cm^−1^; HRMS (FAB): calcd for [C_12_H_15_N_2_O]^+^: 203.1184, found: 203.1178.

## Data Availability Statement

The original contributions presented in the study are included in the article/Supplementary Material, further inquiries can be directed to the corresponding author/s.

## Author Contributions

SL and JY conducted experiments. SY, GL, and DO carried out the collection and analysis of experimental data. MH reviewed and edited the article. SH conducted the initial experiments. H-gP directed the project and wrote the article. All authors contributed to the article and approved the submitted version.

## Conflict of Interest

The authors declare that the research was conducted in the absence of any commercial or financial relationships that could be construed as a potential conflict of interest.
