# Supplementary material for: Enantioselective Synthesis of (+)-Coerulescine by a Phase-Transfer Catalytic Allylation of Diphenylmethyl tert-Butyl α-(2-Nitrophenyl)Malonate
Source: Front Chem. 2020 Nov 12;8:577371. doi: 10.3389/fchem.2020.577371 (PMC7690313; doi:10.3389/fchem.2020.577371)
Supplement: Supplementary file 1 [file Data_Sheet_1.PDF]

Supporting Information

Enantioselective Synthesis of (+)-Coerulescine by Phase-transfer Catalytic Allylation of Diphenylmethyl tert-butyl α-(2-nitrophenyl)malonate

Sanggi Lee,[1] Jewon Yang,[1] Sehun Yang,[1] Geumwoo Lee,[1] Daehyun Oh,[1] Min Woo Ha,[2] Suckchang Hong,[1] and Hyeung-geun Park[1]*

[1]Research Institute of Pharmaceutical Sciences and College of Pharmacy, Seoul National University, Seoul 151-742, Korea, [2]College of Pharmacy, Jeju National University, Seoul 63243, Korea

hgpk@snu.ac.kr

List of Contents

(1) ^{1}H & ^{13}C NMR Spectra -- S2

(2) Chiral HPLC Chromatogram --- S16

(1) ^{1}H & ^{13}C NMR Spectra

^{1}H-NMR of compound **(4)**

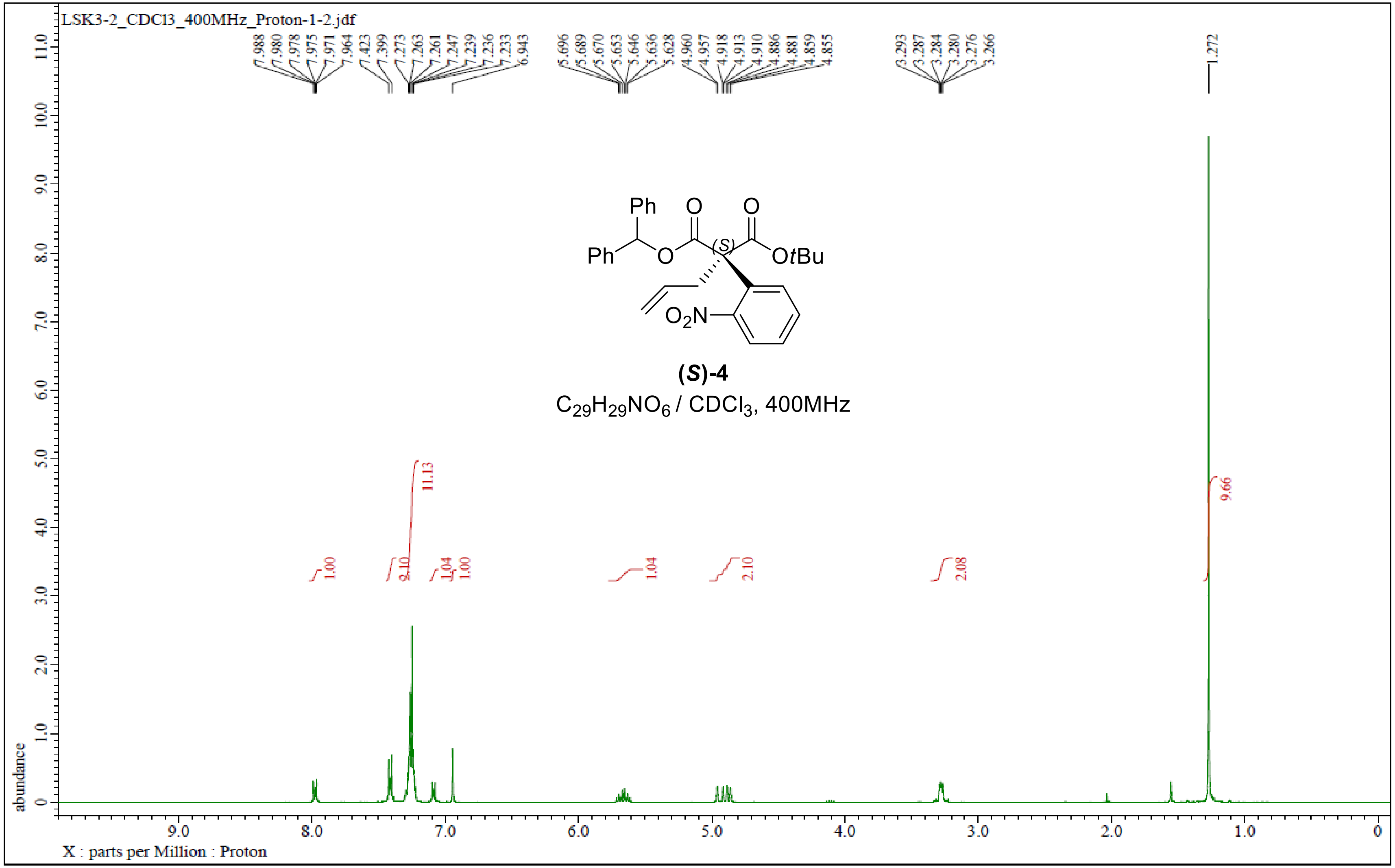

^{13}C-NMR of compound **(4)**

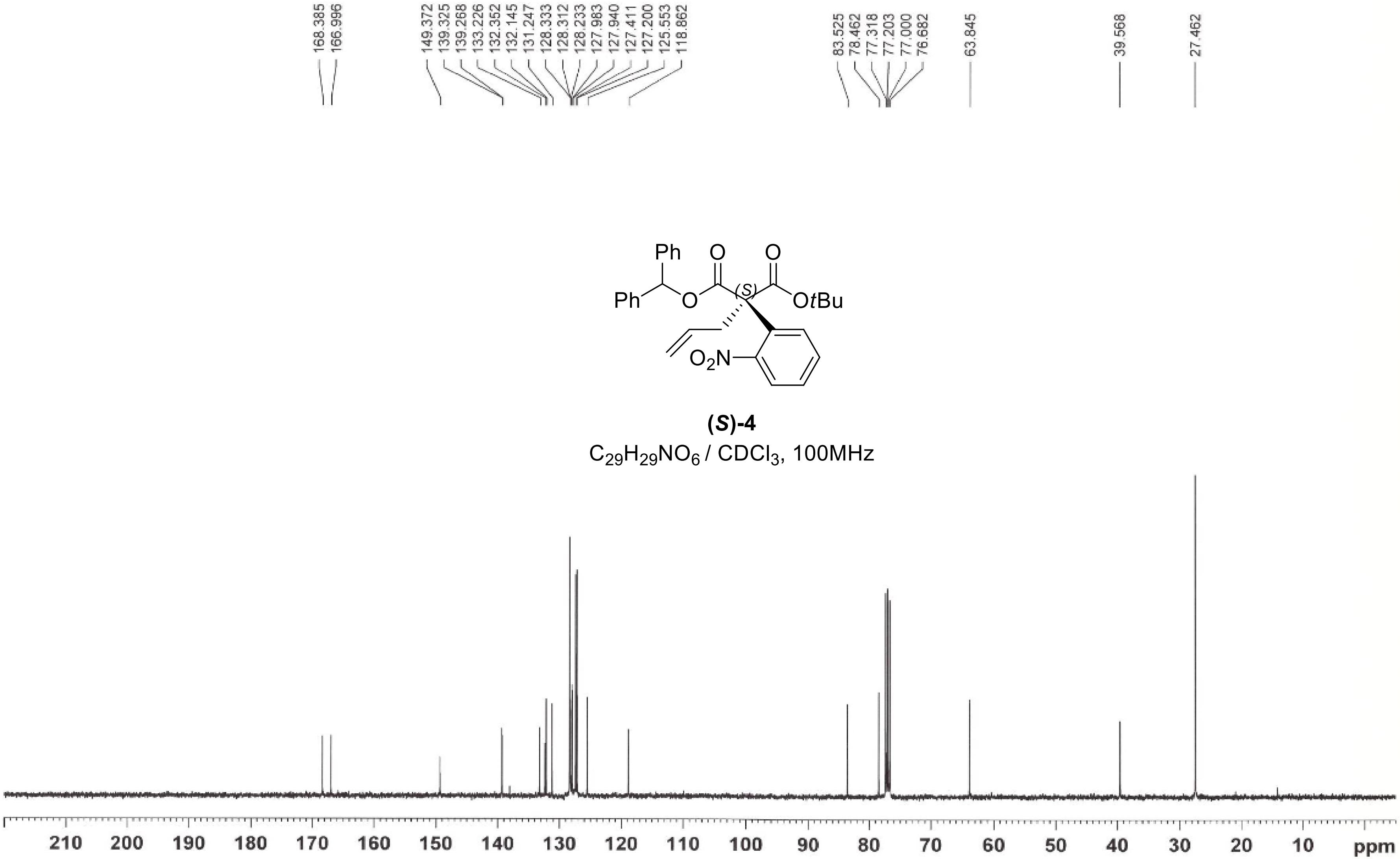

^{1}H-NMR of compound **(5)**

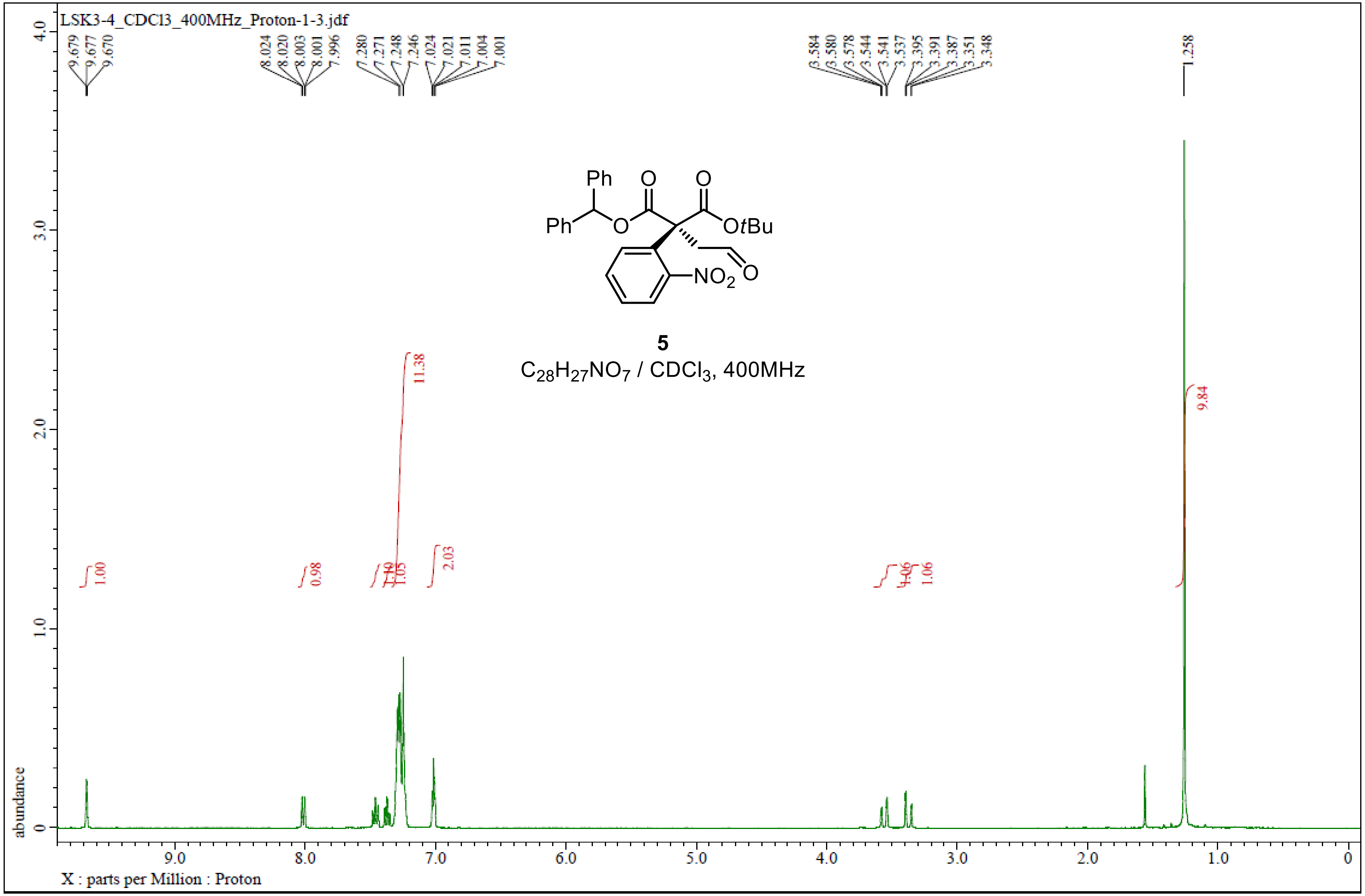

^{13}C-NMR of compound **(5)**

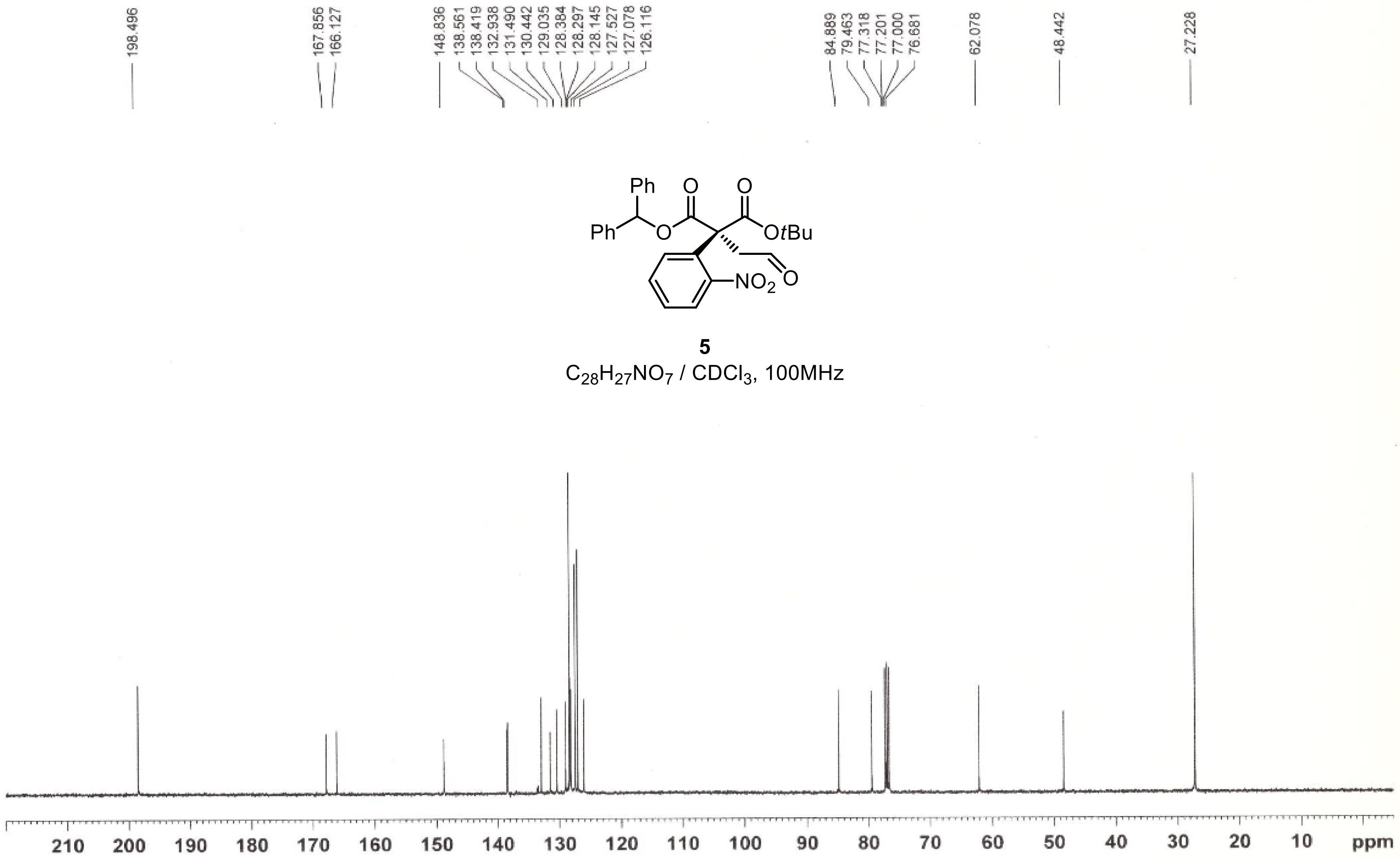

^{1}H-NMR of compound **(6)**

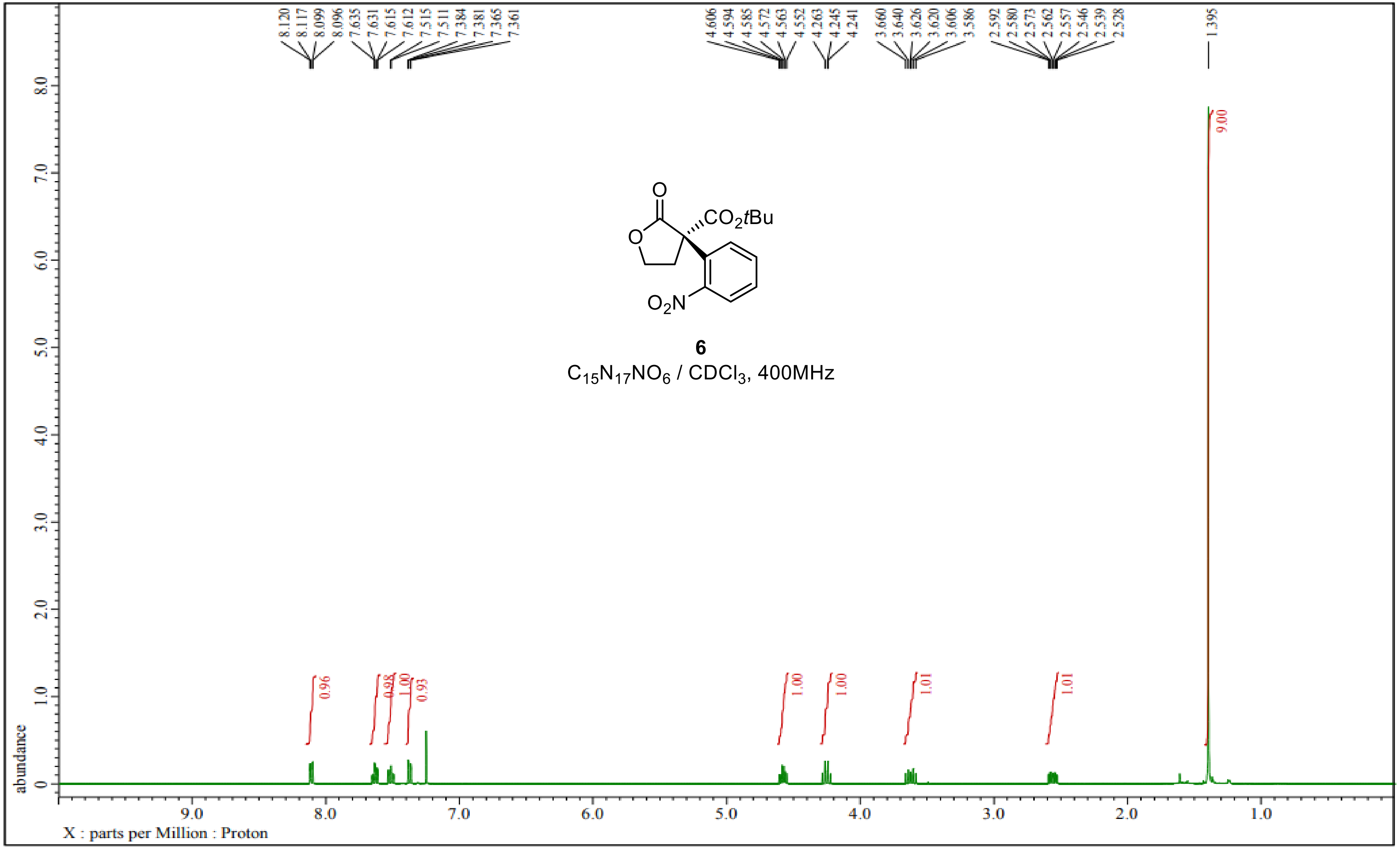

^{13}C-NMR of compound **(6)**

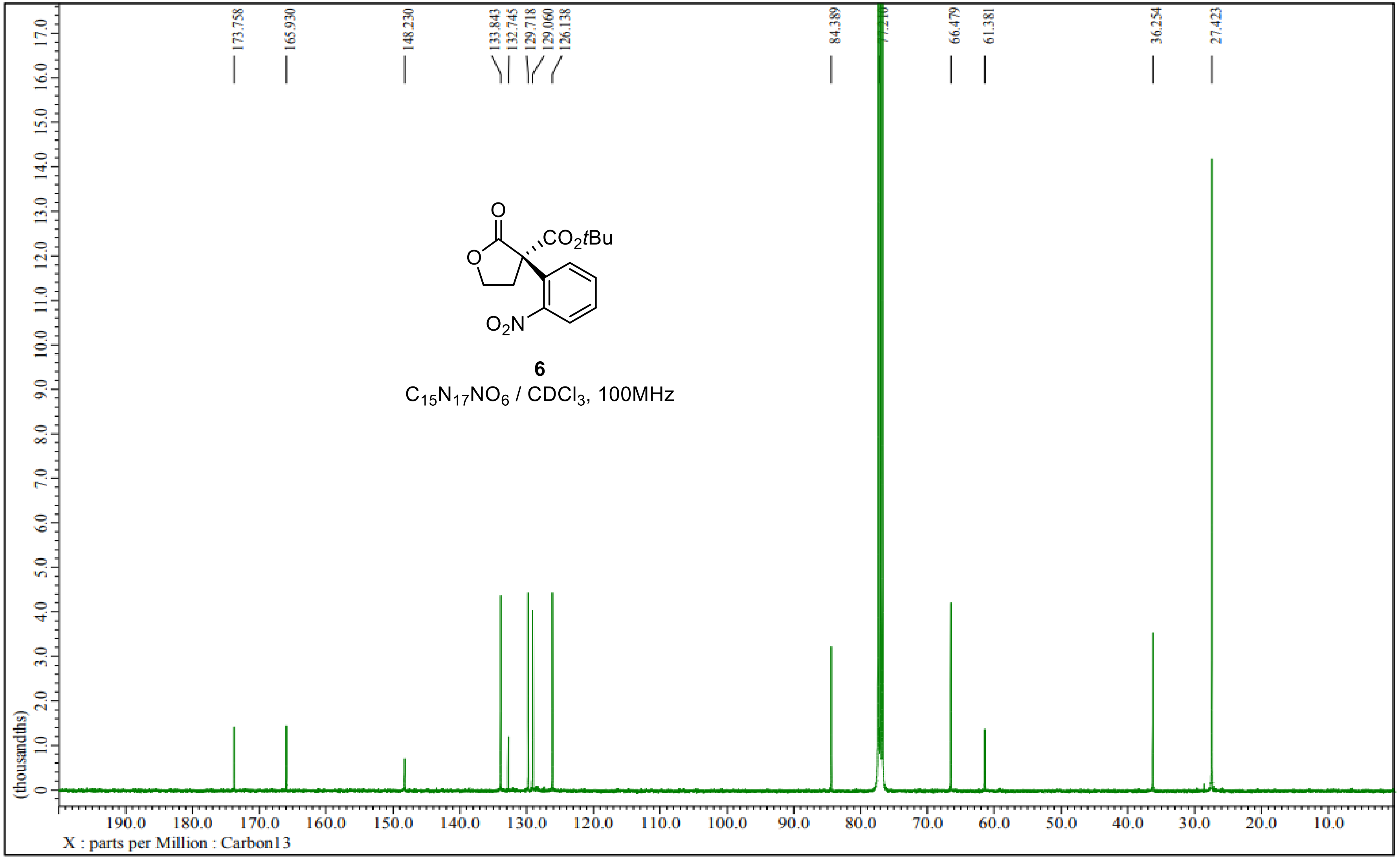

^{1}H-NMR of compound **(7)**

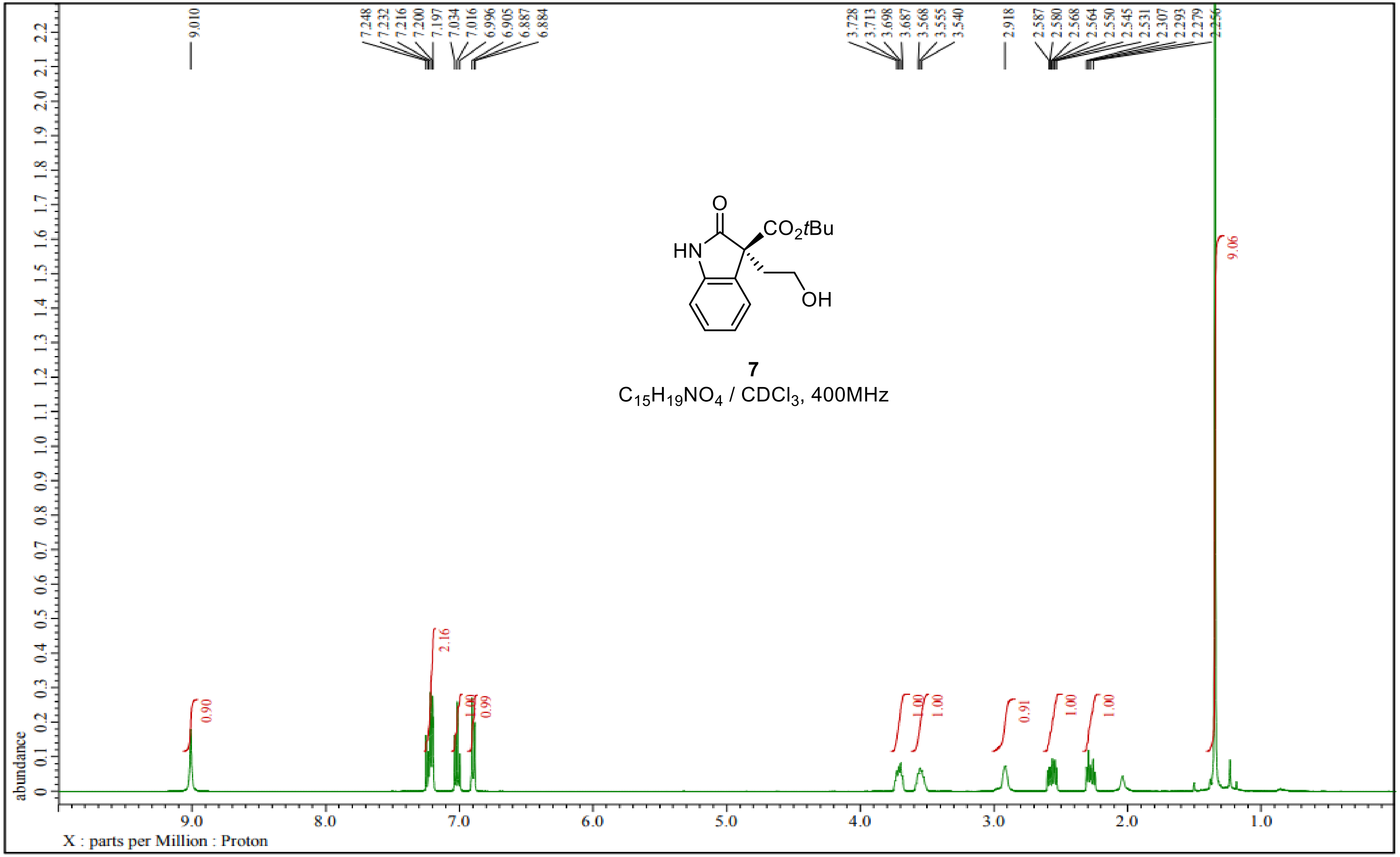

^{13}C-NMR of compound **(7)**

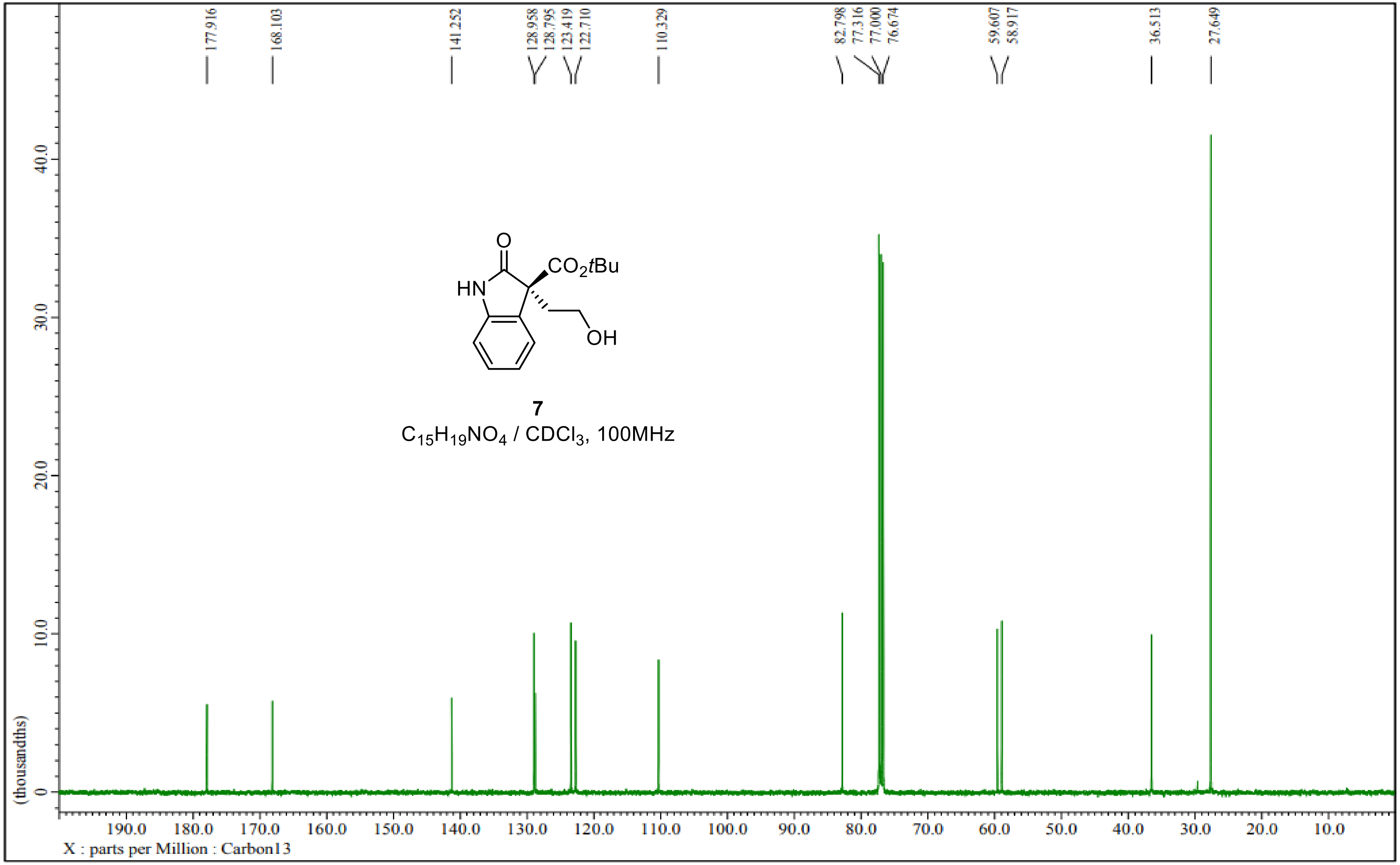

^{1}H-NMR of compound **(8)**

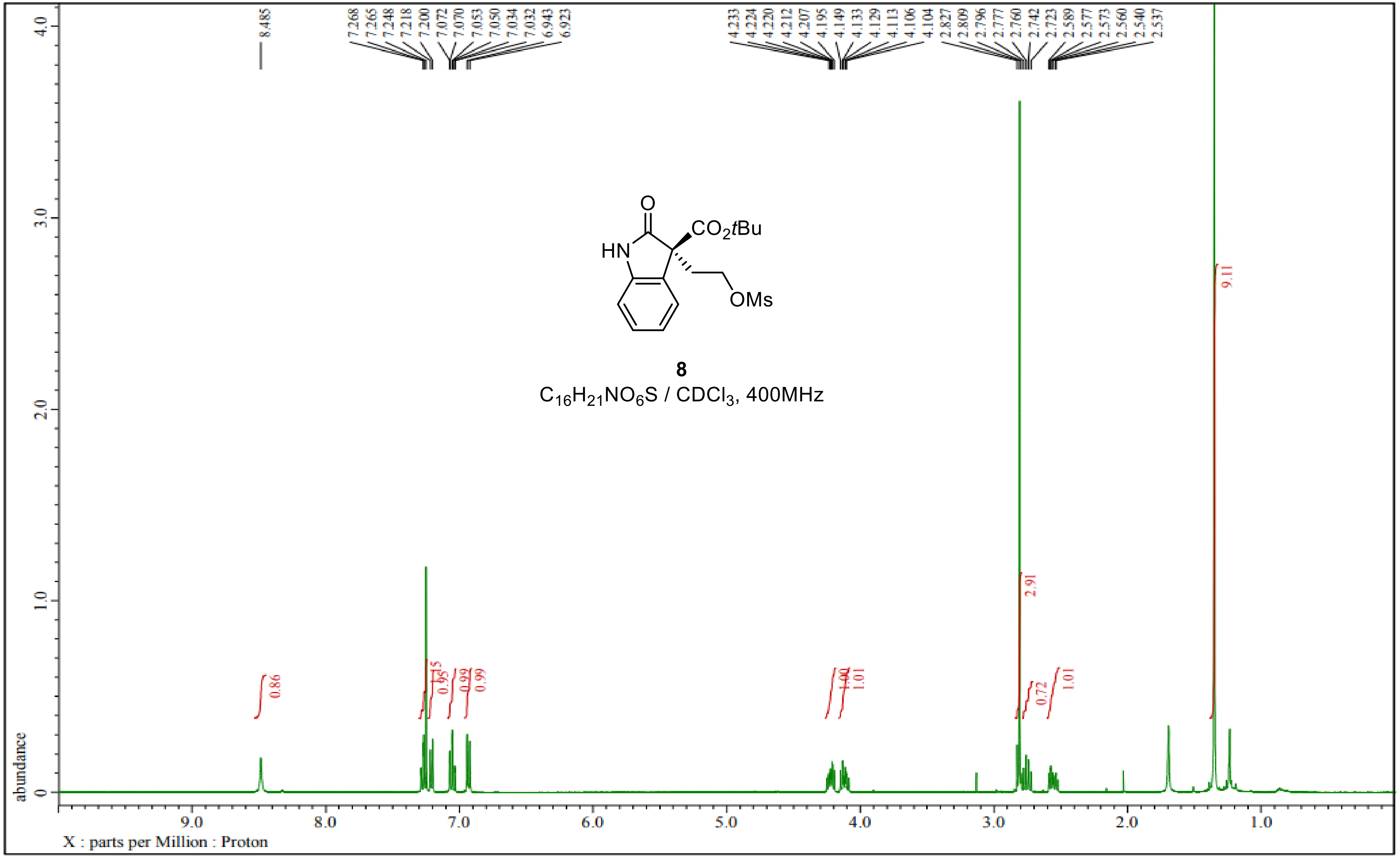

^{13}C-NMR of compound **(8)**

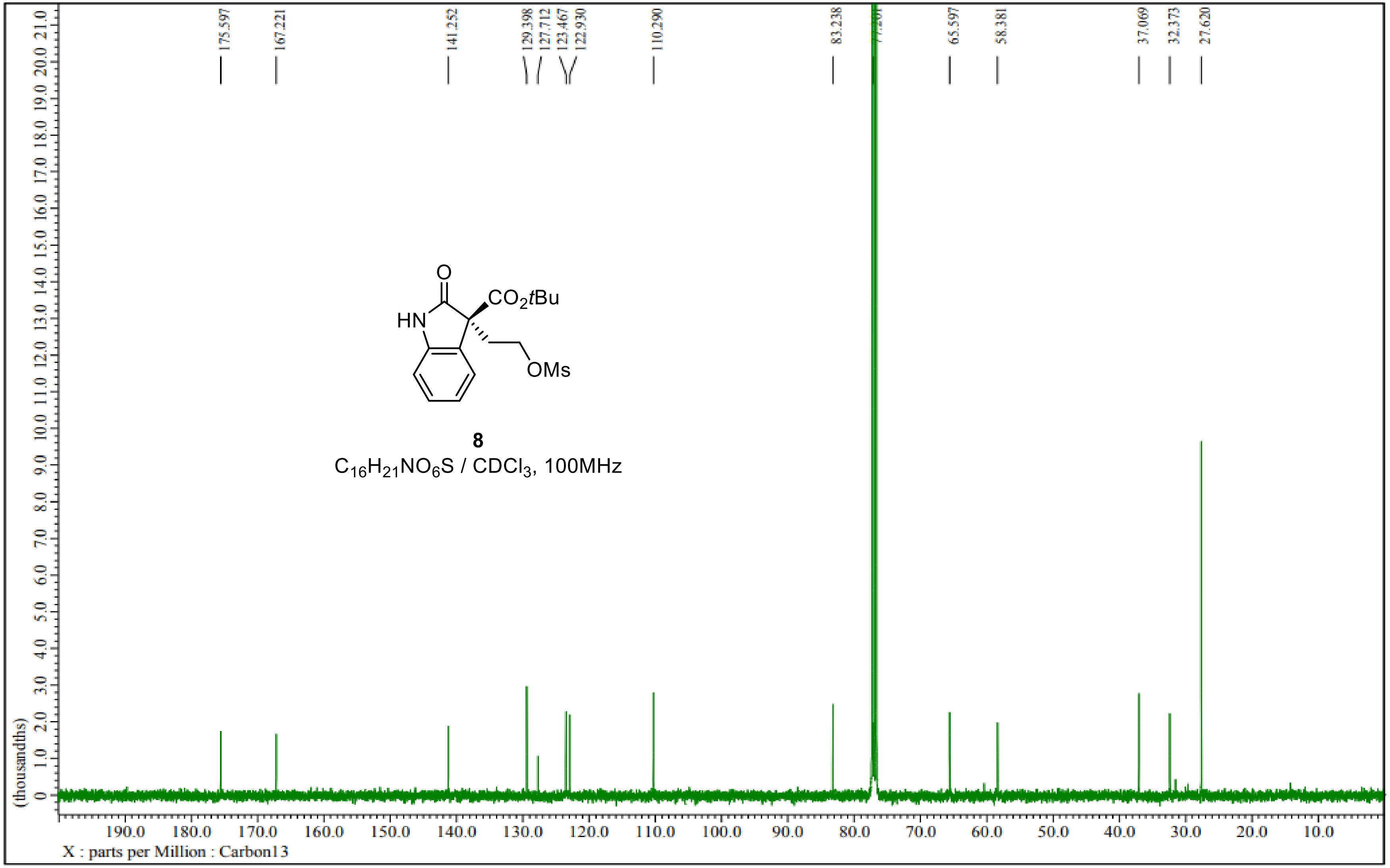

^{1}H-NMR of compound **(10)**

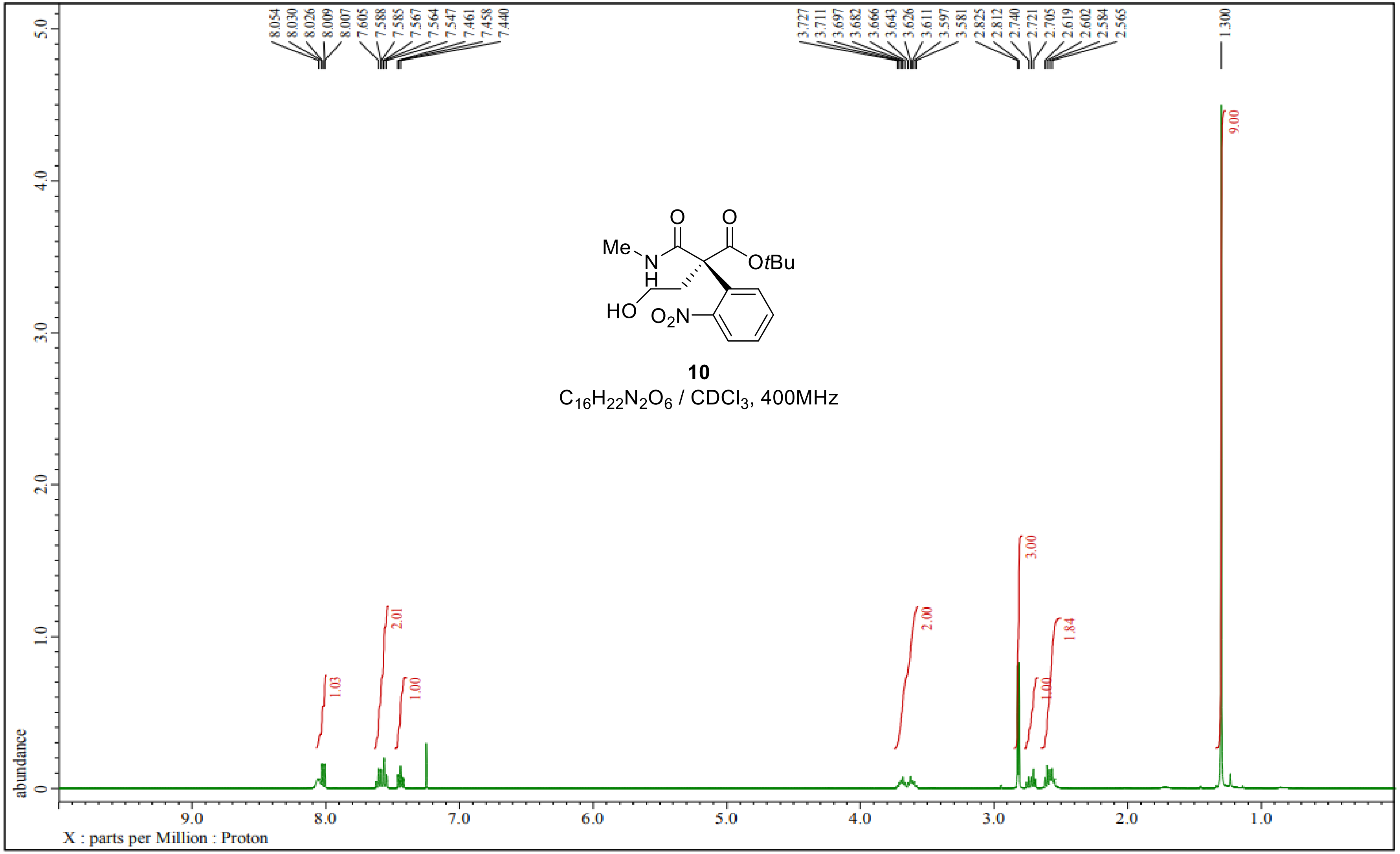

^{13}C-NMR of compound **(10)**

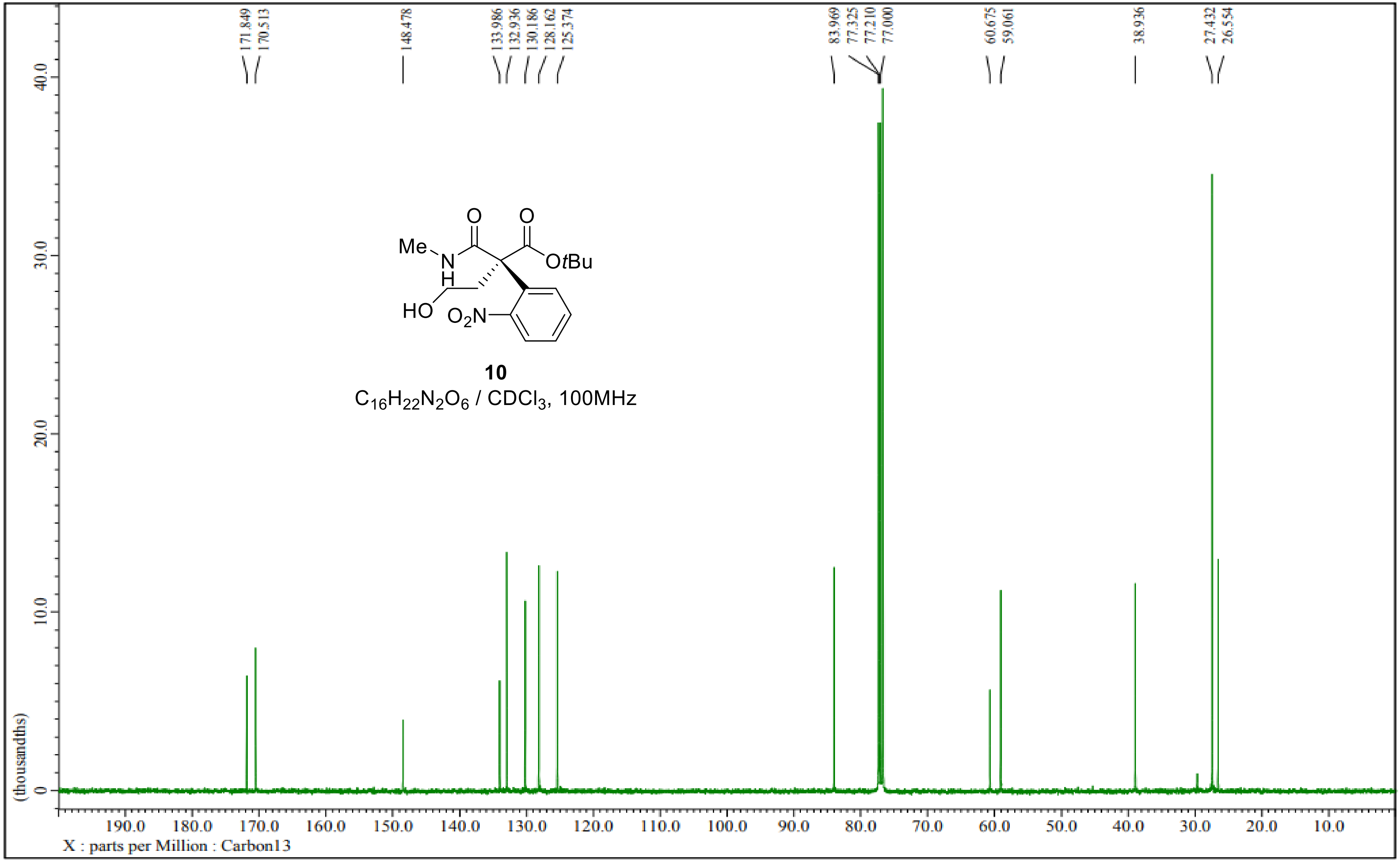

^{1}H-NMR of compound **(11)**

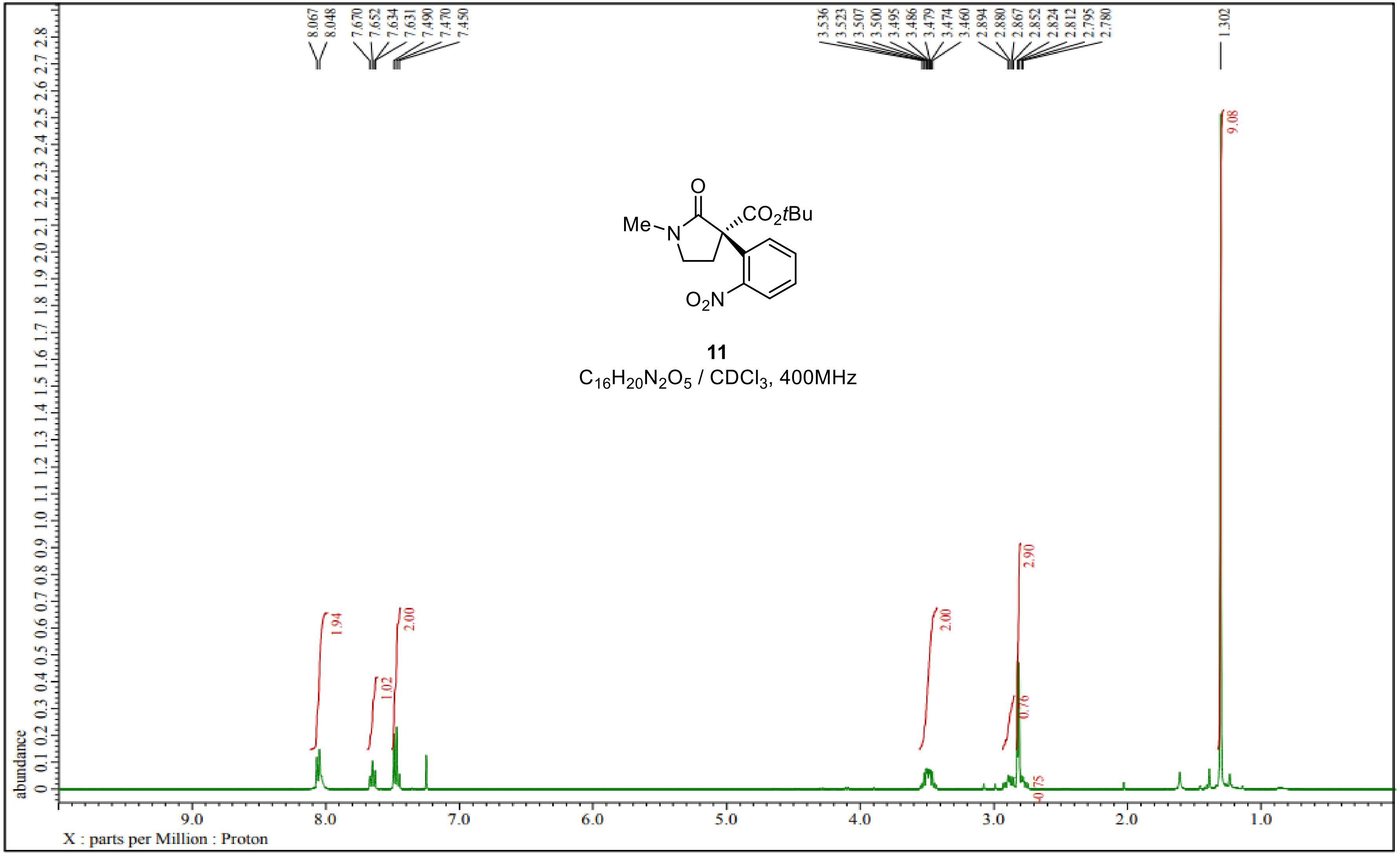

^{13}C-NMR of compound **(11)**

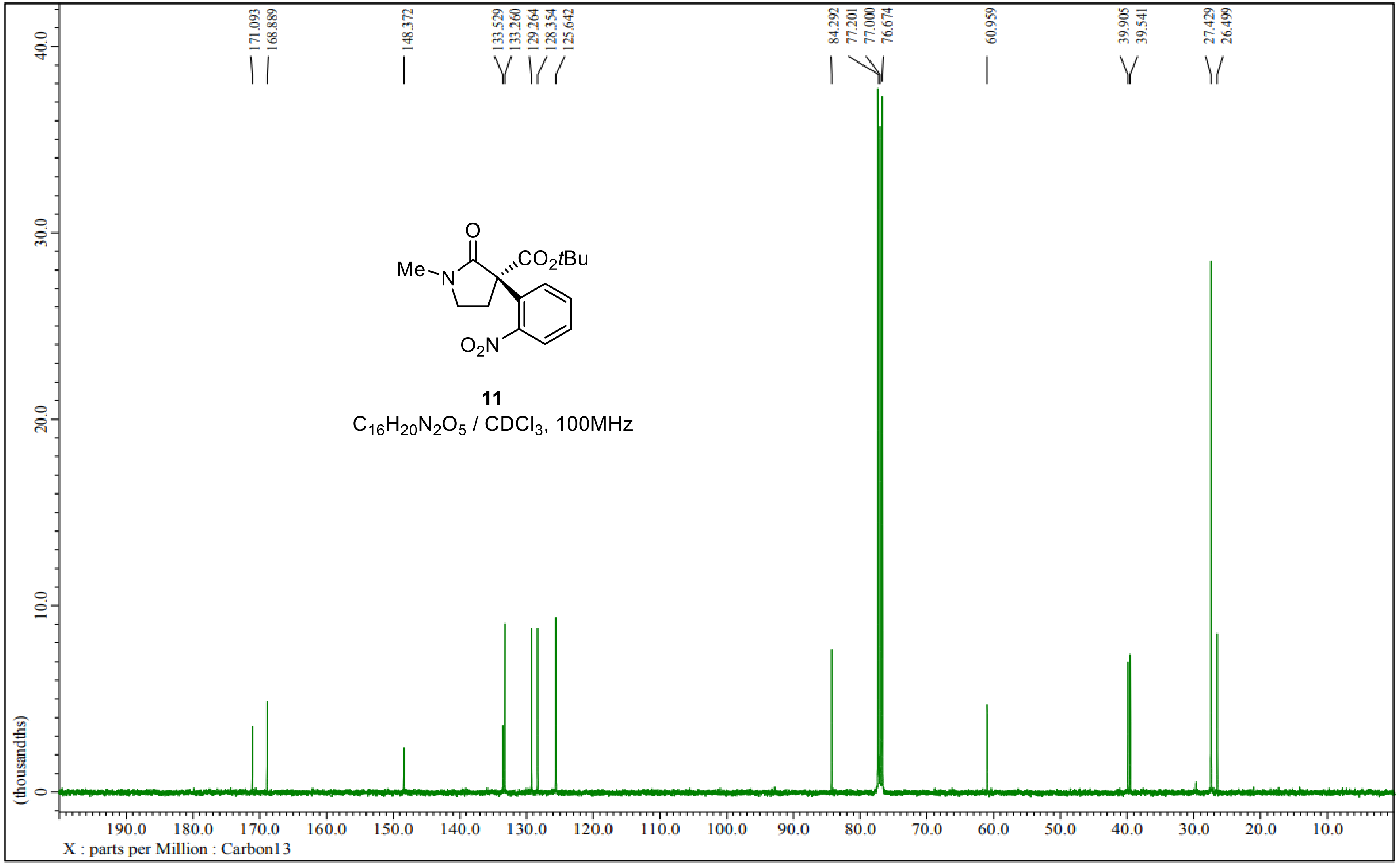

^{1}H-NMR of compound **(13)**

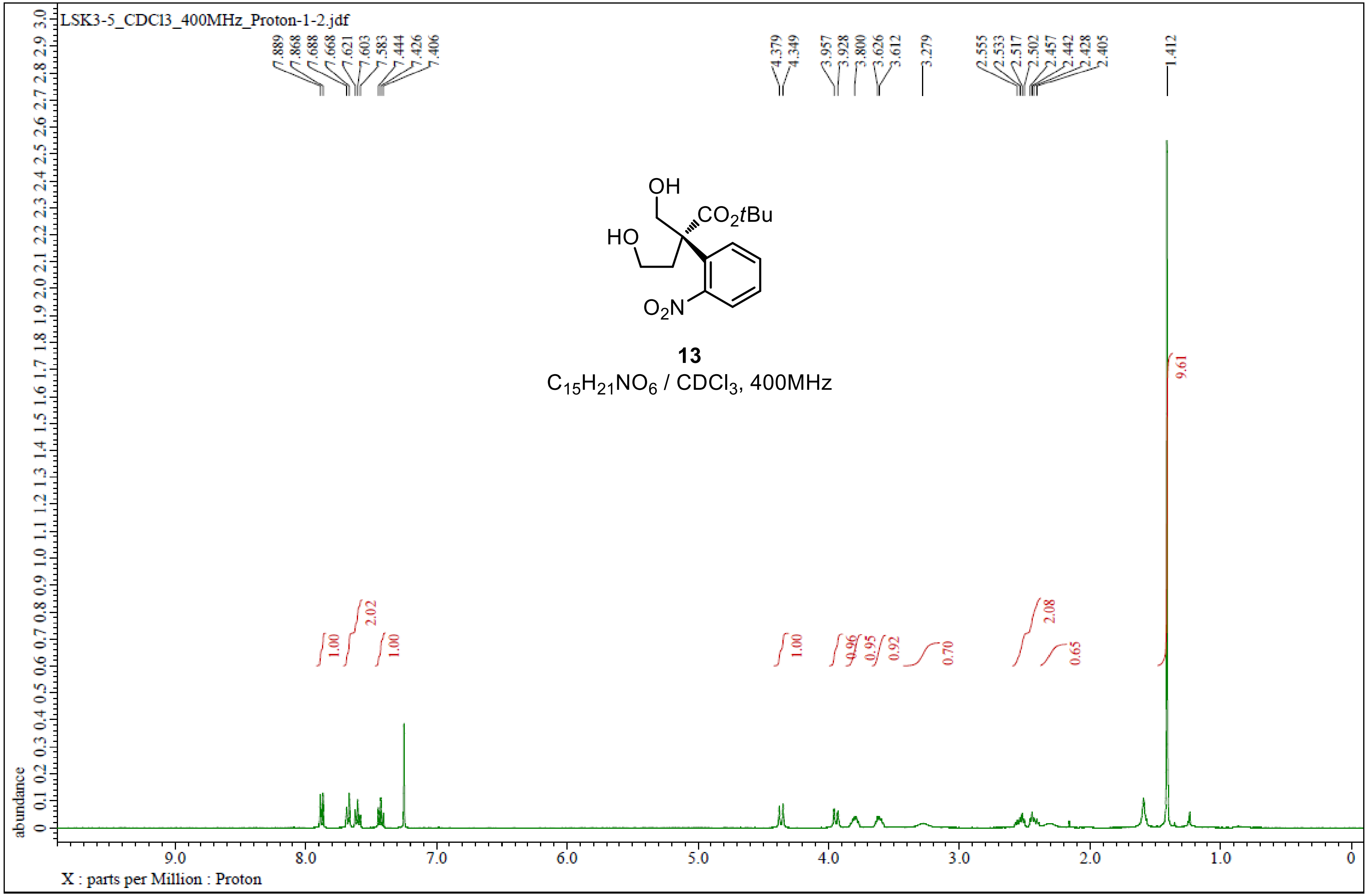

^{13}C-NMR of compound **(13)**

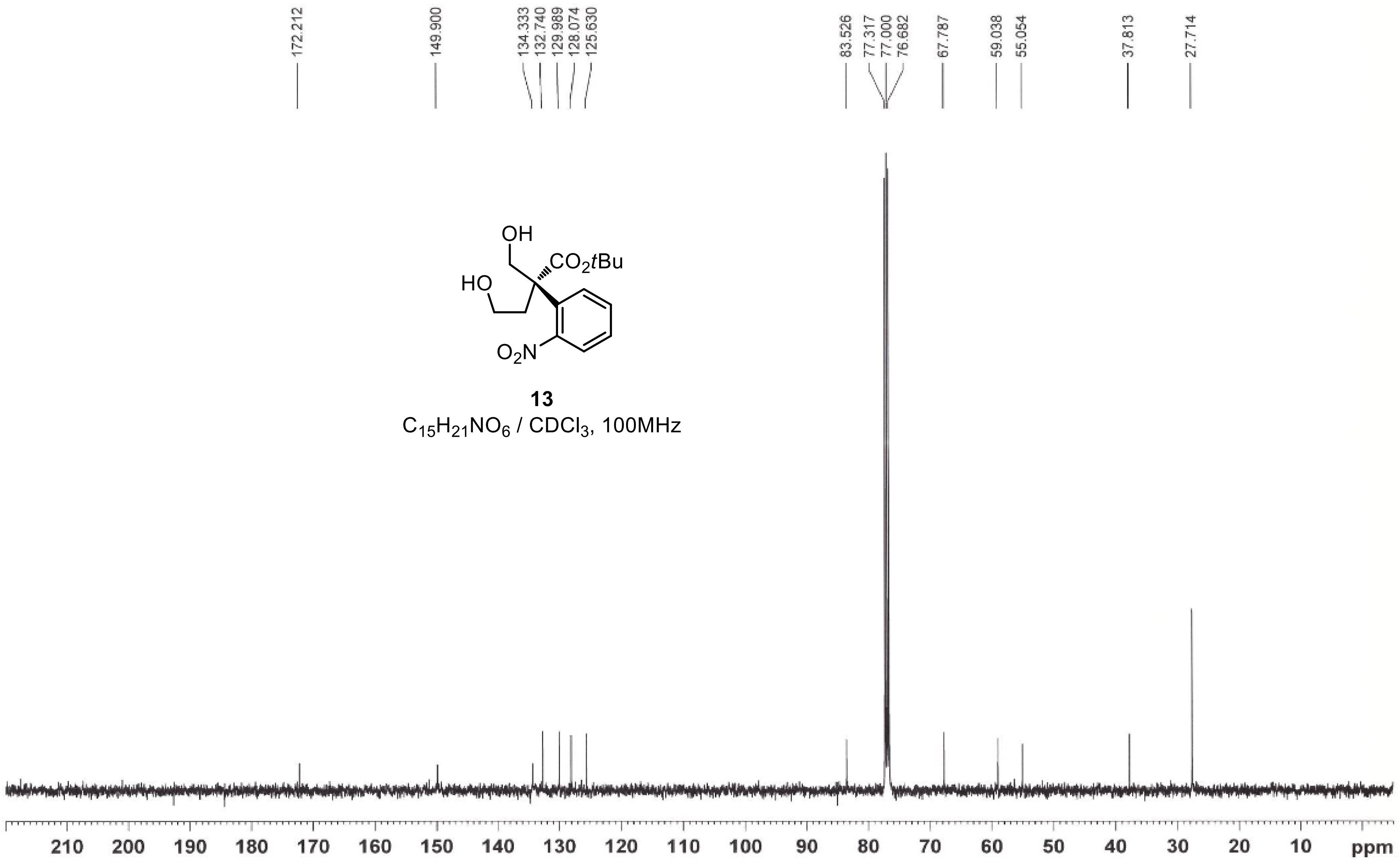

^{1}H-NMR of compound **(14)**

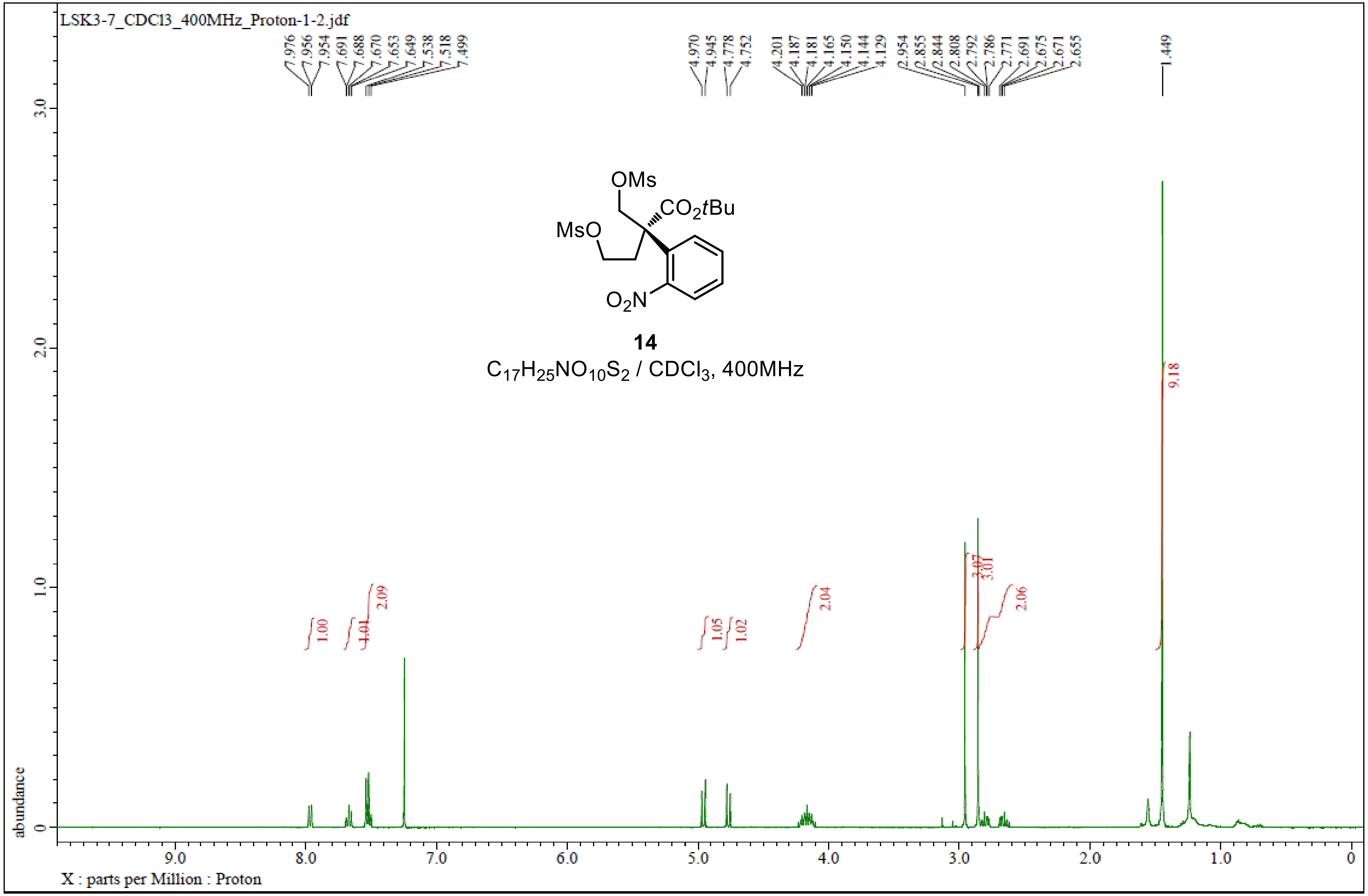

^{13}C-NMR of compound **(14)**

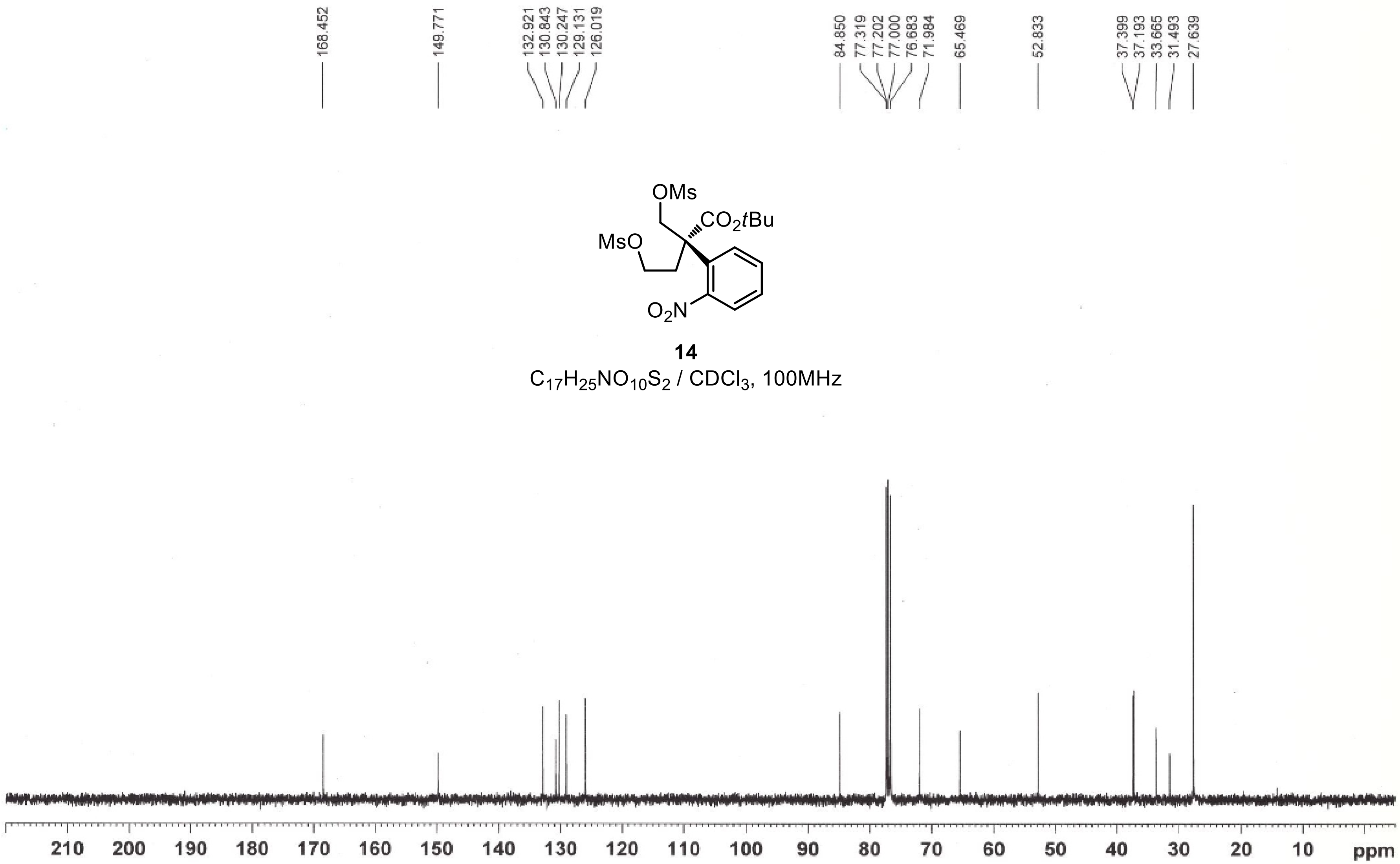

^{1}H-NMR of compound **(15)**

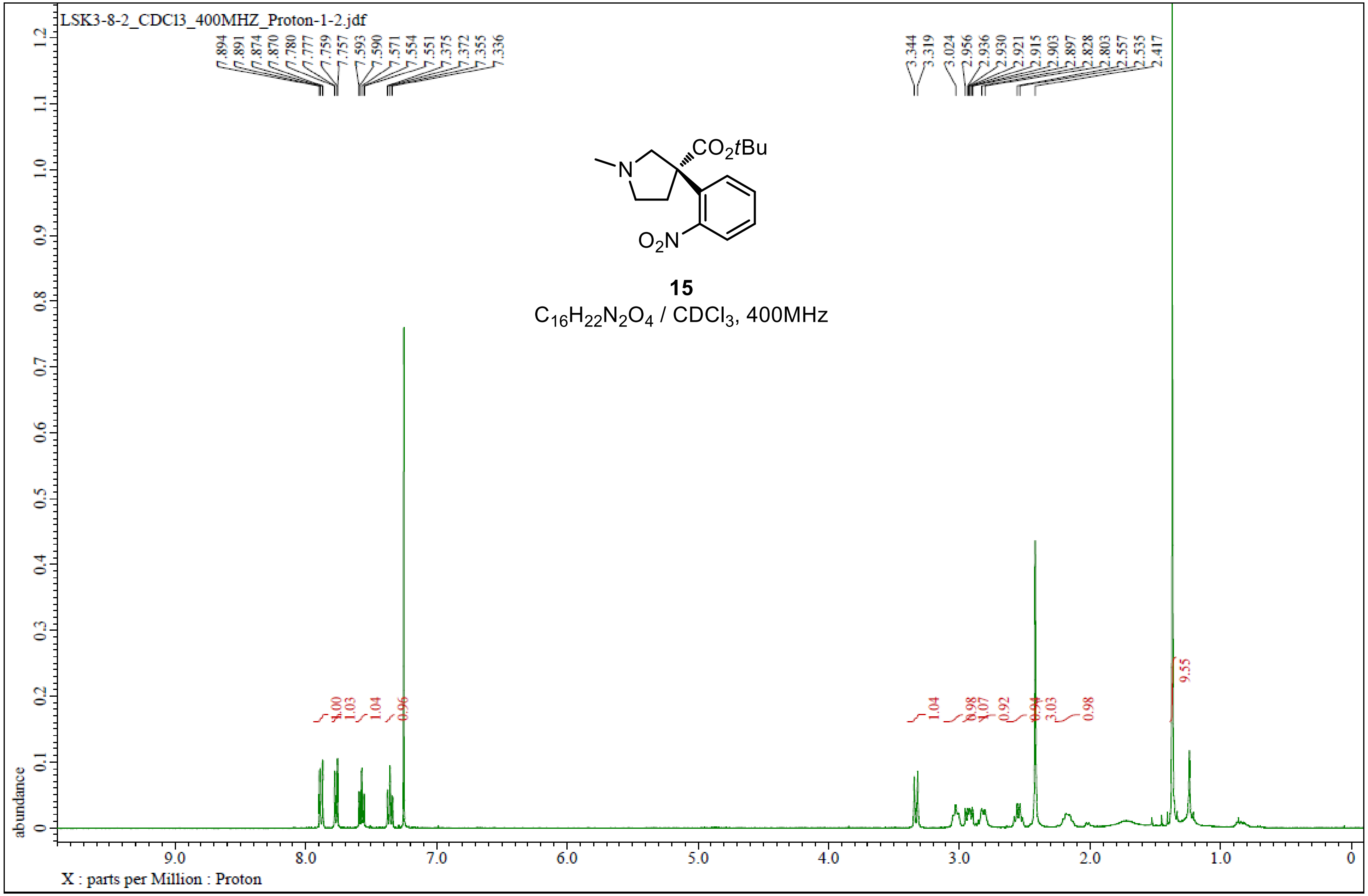

^{13}C-NMR of compound **(15)**

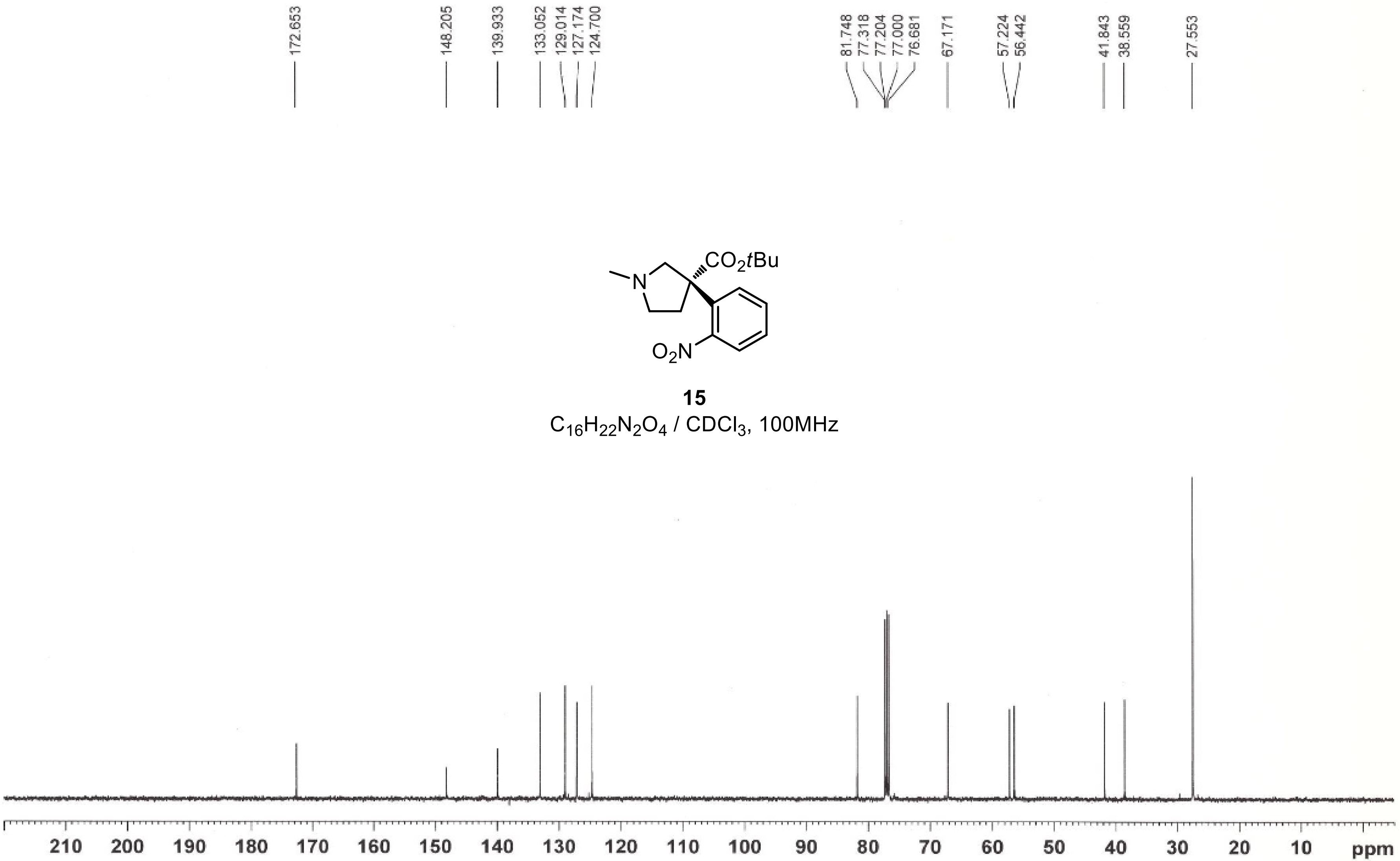

^{1}H-NMR of compound **(+)-coerulescine**

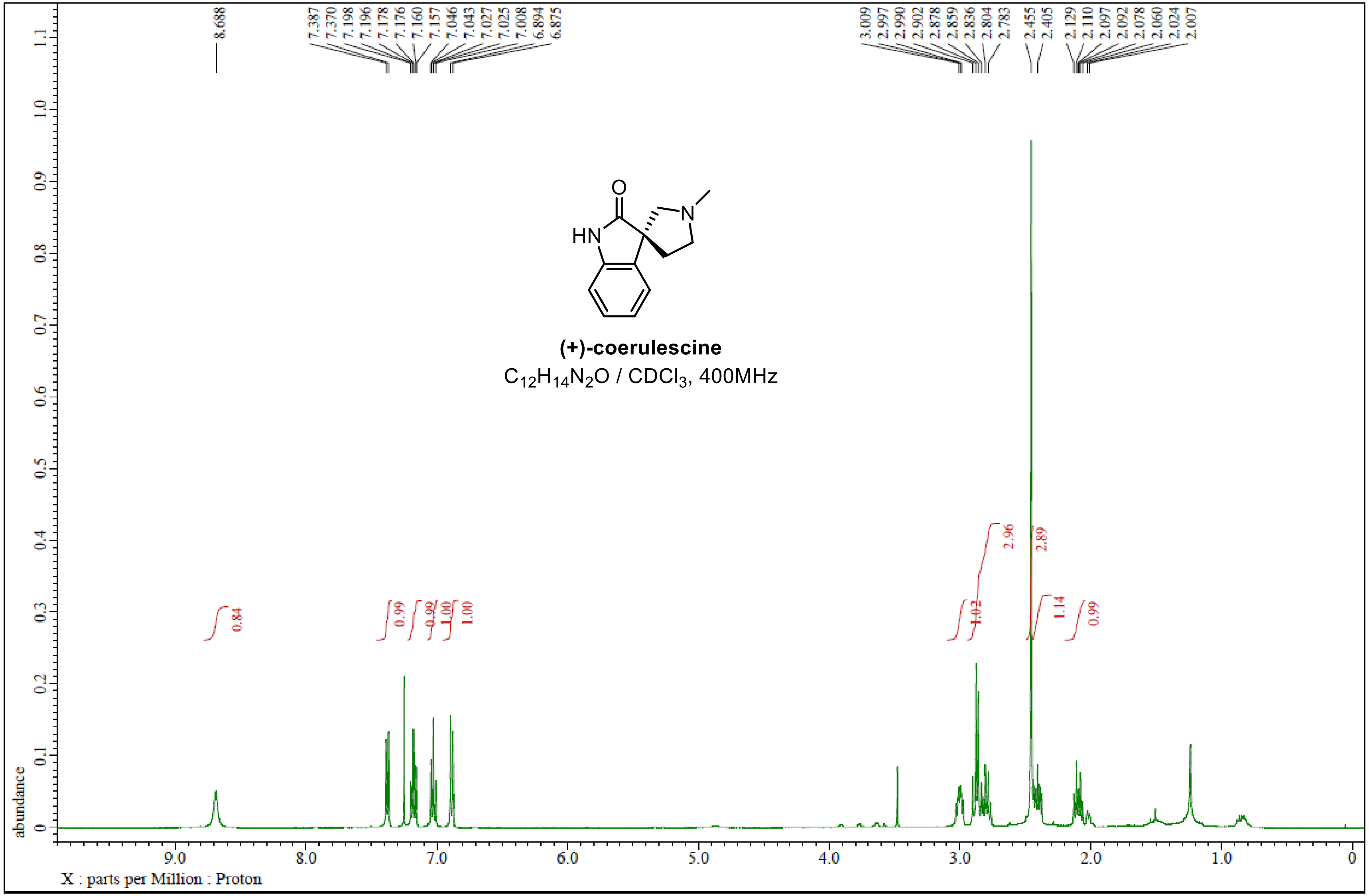

^{13}C-NMR of compound **(+)-coerulescine**

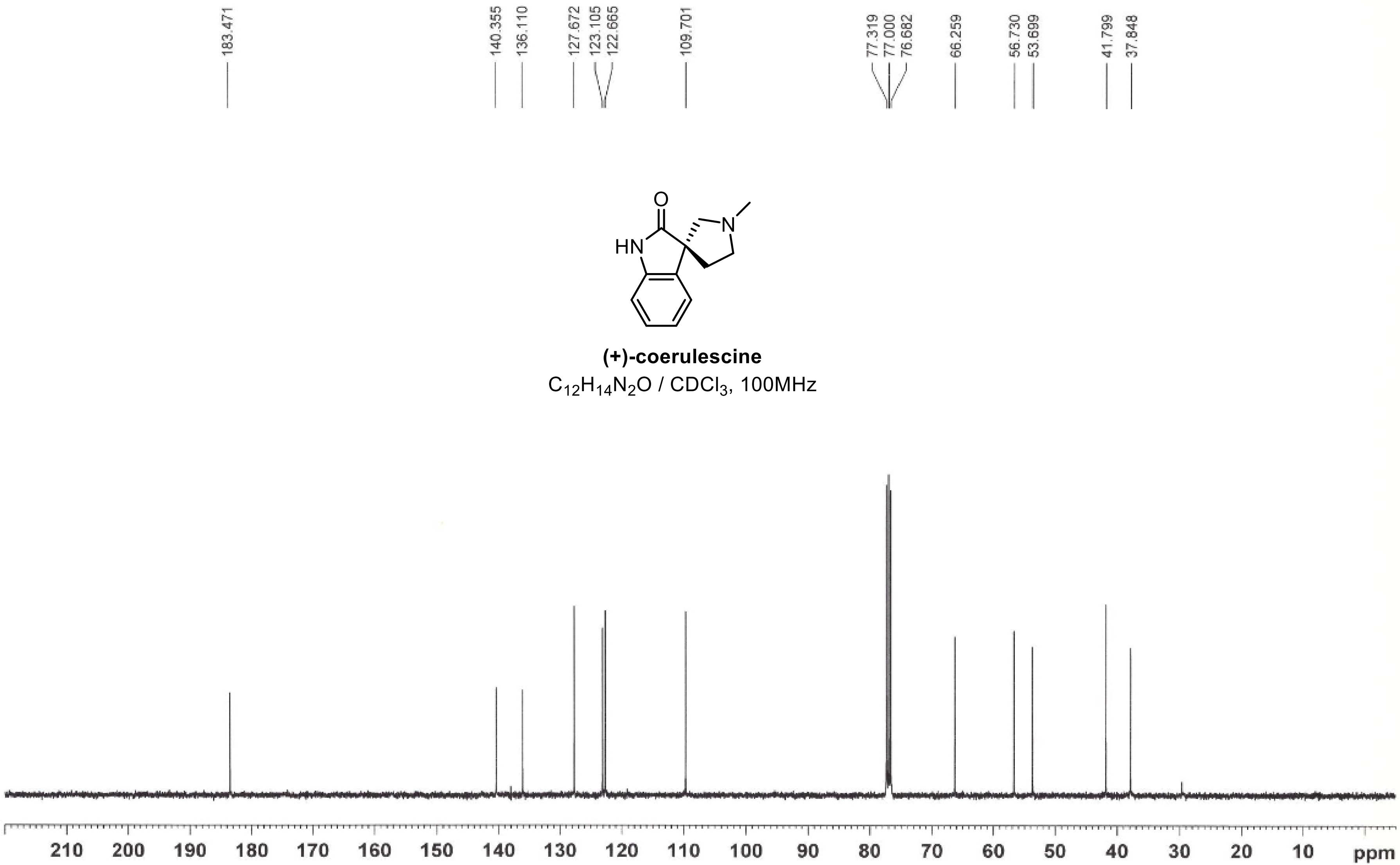

(2) Chiral HPLC spectra

Area Percent Report

Instrument Name : L-2000

Software Version : Version LaChrom 8908800-07

Acquisition Method : Daicel Chiralpak AD-H, Hexane : 2-Propanol = 95:5, λ = 254 nm, flow rate = 1.0 mL/min

Sample ID: *rac*-**5**

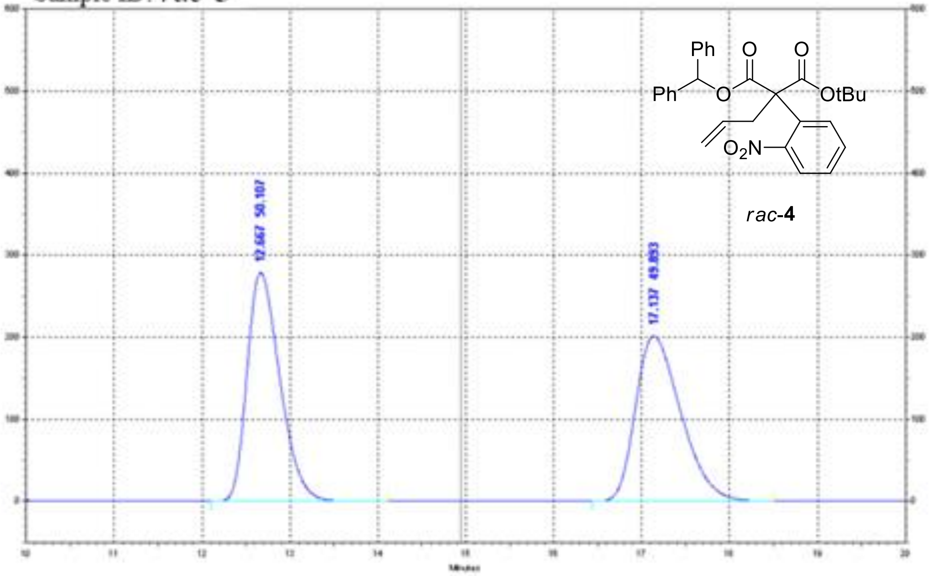

UV Results

Name	Retention Time	Area	Area Percent	Integration Codes
	12.667	28889196	50.107	BI
	17.137	28766122	49.893	BB
Totals		57655318	100.000	

Area Percent Report

Instrument Name : L-2000

Software Version : Version LaChrom 8908800-07

Acquisition Method : Daicel Chiralpak AD-H, Hexane : 2-Propanol = 95:5, λ = 254 nm, flow rate = 1.0 mL/min

Sample ID: *chiral*-**5**

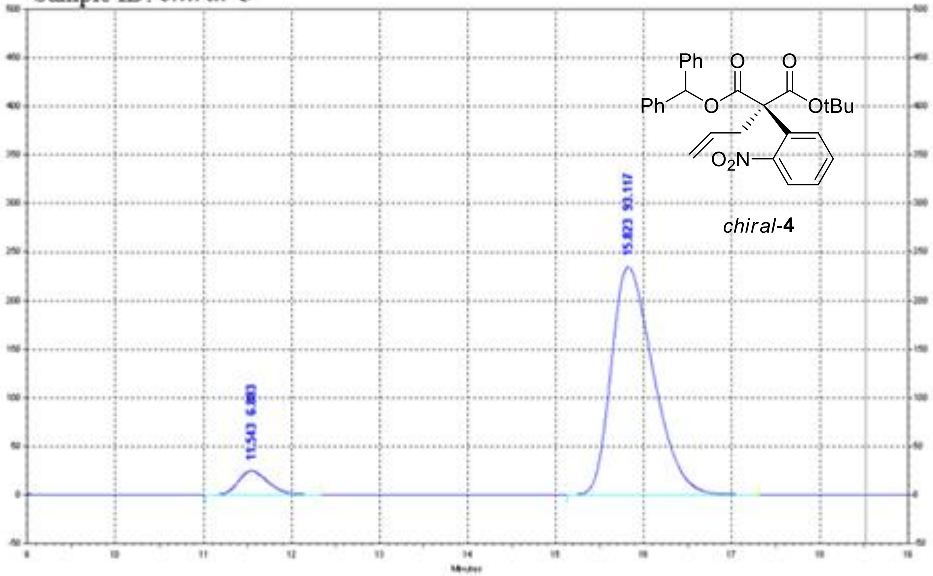

UV Results

Name	Retention Time	Area	Area Percent	Integration Codes
	11.543	2310121	6.883	MM
	15.823	31251904	93.117	MM
Totals		33562025	100.000	

Area Percent Report

Instrument Name : L-2000

Software Version : Version LaChrom 8908800-07

Acquisition Method : Daicel Chiralpak AD-H, Hexane : 2-Propanol = 90:10, λ = 254 nm,

flow rate = 1.0 mL/min

Sample ID: *rac*-**14**

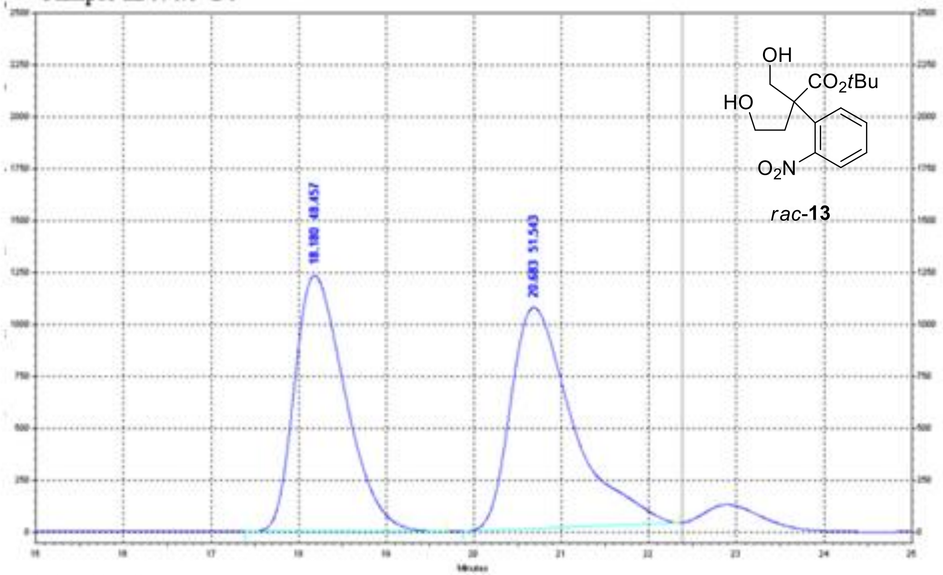

UV Results

Name	Retention Time	Area	Area Percent	Integration Codes
	18.180	198513832	48.457	MM
	20.683	211155480	51.543	MM
Totals		409669312	100.000	

Area Percent Report

Instrument Name : L-2000

Software Version : Version LaChrom 8908800-07

Acquisition Method : Daicel Chiralpak AD-H, Hexane : 2-Propanol = 90:10, λ = 254 nm,

flow rate = 1.0 mL/min

Sample ID: *chiral*-**14**

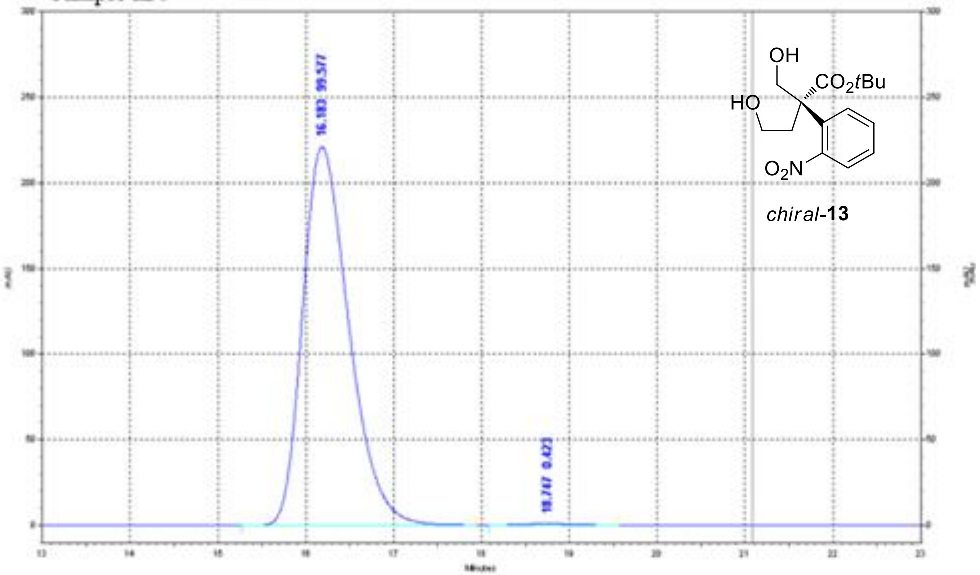

UV Results

Name	Retention Time	Area	Area Percent	Integration Codes
	16.183	32374002	99.577	MM
	18.747	137537	0.423	MM
Totals		32511539	100.000	

Area Percent Report

Instrument Name : L-2000

Software Version : Version LaChrom 8908800-07

Acquisition Method : Daicel Chiralpak AS-H, Hexane : 2-Propanol = 90:10, λ = 254 nm, flow rate = 1.0 mL/min

Sample ID: **(±)-coerulescine**

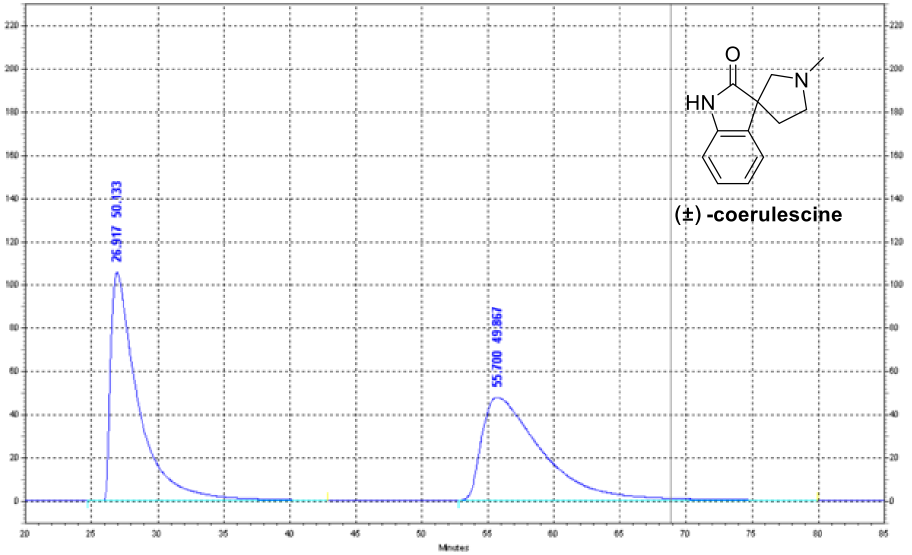

UV Results

Name	Retention Time	Area	Area Percent	Integration Codes
	26.917	60791357	50.133	MM
	55.700	60468972	49.867	MM
Totals		121260329	100.000	

Area Percent Report

Instrument Name : L-2000

Software Version : Version LaChrom 8908800-07

Acquisition Method : Daicel Chiralpak AS-H, Hexane : 2-Propanol = 90:10, λ = 254 nm, flow rate = 1.0 mL/min

Sample ID: **(+)-coerulescine**

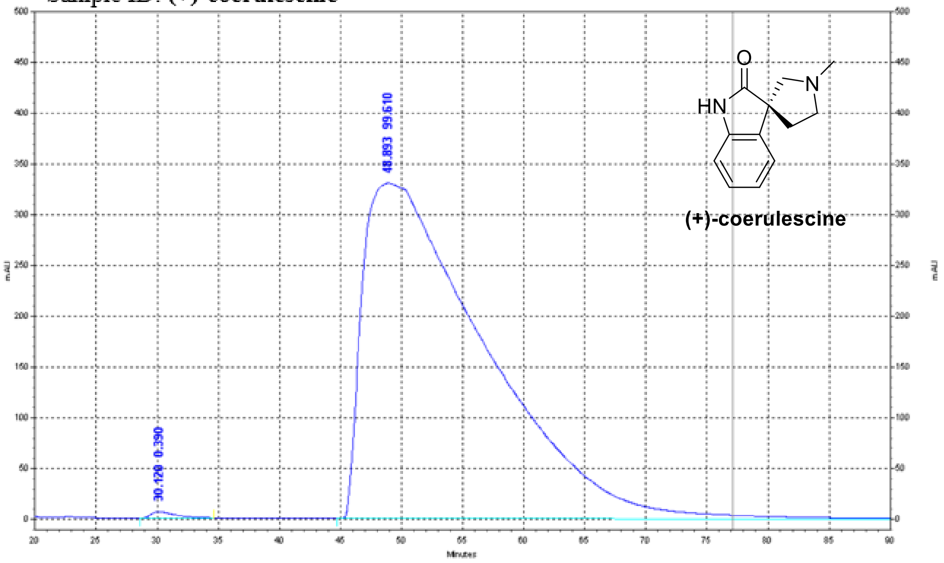

UV Results

Name	Retention Time	Area	Area Percent	Integration Codes
	30.120	3610356	0.390	MM
	48.893	921745041	99.610	MM
Totals		925355397	100.000	
